# Social determinants in the access to health care for Chagas disease: A qualitative research on family life in the “Valle Alto” of Cochabamba, Bolivia

**DOI:** 10.1371/journal.pone.0255226

**Published:** 2021-08-12

**Authors:** I. Jimeno, N. Mendoza, F. Zapana, L. de la Torre, F. Torrico, D. Lozano, C. Billot, M. J. Pinazo, on behalf of the InSPIRES Consortium

**Affiliations:** 1 Barcelona Institute for Global Health (ISGlobal), Barcelona, Spain; 2 Fundación Ciencia y Estudios Aplicados para el Desarrollo en Salud y Medio Ambiente (CEADES Foundation), Cochabamba, Bolivia; Tulane University, UNITED STATES

## Abstract

**Introduction:**

Chagas disease is caused by the *Trypanosoma cruzi* infection. It is a neglected tropical disease with considerable impact on the physical, psychological, familiar, and social spheres. The Valle Alto of Cochabamba is a hyperendemic region of Bolivia where efforts to control the transmission of the disease have progressed over the years. However, many challenges remain, above all, timely detection and health-care access.

**Methods:**

Following the Science Shop process, this bottom-up research emerged with the participation of the civil society from Valle Alto and representatives of the Association of *Corazones Unidos por el Chagas* from Cochabamba. The aim of this study is to explore the social determinants in the living realities of those affected by Chagas disease or the silent infection and how families in the Valle Alto of Cochabamba cope with it. An interdisciplinary research team conducted a case study of the life stories of three families using information from in-depth interviews and performed a descriptive qualitative content analysis and triangulation processes.

**Findings:**

Findings provide insights into social circumstances of the research subjects’ lives; particularly, on how exposure to *Trypanosoma cruzi* infection affects their daily lives in terms of seeking comprehensive health care. Research subjects revealed needs and shared their experiences, thus providing an understanding of the complexity of Chagas disease from the socioeconomic, sociocultural, political, and biomedical perspectives. Results enlighten on three dimensions: structural, psychosocial, and plural health system. The diverse perceptions and attitudes toward Chagas within families, including the denial of its existence, are remarkable as gender and ethnocultural aspects. Findings support recommendations to various stakeholders and translation materials.

**Conclusions:**

Intersectional disease management and community involvement are essential for deciding the most appropriate and effective actions. Education, detection, health care, and social programs engaging family units ought to be the pillars of a promising approach.

## Introduction

### Introduction to Chagas disease

Chagas disease, a parasitic infection caused by the protozoan *Trypanosoma cruzi* *(T. cruzi)*, affects about six million people worldwide [1]. It is estimated that 70 million people are at risk of contracting the infection [1]. It mostly appears in the continental areas of 21 American countries where multiple hematophagous Hemiptera species (Fam. Reduvidae) may transmit the parasite through their feces. However, oral transmission through food or drinks contaminated by the vector rarely causes epidemic outbreaks [2]. Other globally relevant forms of transmission include vertical transmission, blood transfusion, and organ transplantation [1]. This vector-borne disease is historically associated with situations of vulnerability and precariousness prevalent among many people living in endemic rural areas of Latin American countries [3]. Therefore, despite its discovery more than a century ago, it is still a disease of minor interest and ignored, being among the 20 neglected diseases listed by the World Health Organization (WHO) [4]. With the increase in international migration, Chagas disease can now be traced in many other non-endemic areas and countries [5].

*Trypanosoma cruzi* infection develops in two phases. In the acute phase, most cases are asymptomatic or experiment mild non-specific symptoms, which hinders timely detection. Nonetheless, severe symptoms may appear in the acute phase in cases with a high level of parasitemia, as can be observed in vertical and oral transmissions or under immunosuppressive conditions [6, 7]. If the infection remains untreated, it progresses to a chronic phase, often in an indeterminate form without apparent clinical manifestations. However, 40% of those infected will suffer from a cardiac, digestive, or eventually neurological pathology or a combination of them, usually within 10–20 years after the time of infection [7]. Thus, due to the absence of symptoms, many people are unaware of the infection by *T. cruzi*. However, as the pathology progresses, organ damages are irreversible and may cause high morbidity or death. It is estimated that there are 14,000 deaths each year worldwide and an annual global burden of 800,000 disability-adjusted life years [1, 8]. This disabling, fatal, and stigmatizing disease highly compromises the quality of life of those affected, with psychological, social, and economic implications [9–12]. On the other hand, it also has a high socioeconomic impact on public systems, involving global health costs of about US$ 7.2 billion per year [8].

Two trypanocidal drugs were discovered in the 1970s: Benznidazole (RO 7–1051) [13] and Nifurtimox (Bayer 2502) [14]. However, their use was discouraged in the past because there was no scientific consensus on their effectiveness and on the involvement of the autoimmunity mechanism in the emergence of pathologies. Therefore, patients were diagnosed with a fatal, incurable, and unmanageable disease. This knowledge and this medical practice have been considered the old paradigm that precedes the current evidence and recommendations [15]. Today, etiological treatment has been shown to be highly effective in eliminating the parasite or containing the infection and is recommended by the Pan American Health Organization (PAHO) in acute phases, newborns, pediatric ages, and in early chronic indeterminate phases [16, 17]. In addition, women of childbearing age benefit from etiological treatment to prevent vertical transmission [18, 19]. However, there are still discrepancies about its use in the chronic phase for patients who have already developed chagasic cardiac complications [17, 20]. Besides, drugs are contraindicated for patients with specific comorbidities and often cause Adverse Drug Reactions (ADRs) that prevent the completion of the two-month regimen [21–23]. As no biomarkers of therapeutic efficacy have been found [24], patients require long-term or lifetime follow-up. Despite some limitations, the effectiveness and tolerance of the etiological treatment of chronic *T. cruzi* infection have been demonstrated (Class IIa recommendation, Level of Evidence B), with a lower mortality, a decrease of severe heart conditions, and a complete or partial seronegative conversion that corresponds to a reduction in the parasitic load [25–28]. Nevertheless, timely detection is essential to ensure the effectiveness of the etiological treatment. Thus, although trypanocidal drugs are included in the WHO List of Essential Medicines [29, 30], less than 1% of people with *T. cruzi* worldwide access treatment [31, 32].

The best-known obstacles are [10, 33–36]: i) low priority in political and health systems, ii) low knowledge and awareness, even among health personnel, iii) low demand for diagnostic and therapeutic products, which results in low production, low availability, and high costs, iv) diagnostic and therapeutic challenges, v) social inequalities, and vi) stigmatization of Chagas disease.

Increasing access to comprehensive health care is a central axis and a priority for improving the health of people living with *T. cruzi*, reducing transmission circuits, and decreasing social and public health costs. However, overcoming the complexity of Chagas disease integrally is still a long road ahead. In this respect, the analysis of the social determinants of Chagas disease is key for understanding the inequalities in exposure and the impact of the disease on several population groups [32, 37].

### Historical overview of Chagas disease in Bolivia

Chagas disease was discovered and described in 1909 by Carlos Ribeiro Justiniano das Chagas in Brazil and by Salvador Mazza in Argentina, but it was not until the 1940s when the first cases of Chagas disease were reported in Bolivia [38]. Moreover, the prevalence studies and vector control campaigns in Bolivia began in the 1980s, with the support of international Non-Governmental Organizations (NGOs) [39]. In 1986, the Regional Chagas Tupiza-Cotagaita Program for the Comprehensive Control of Chagas Disease was initiated. This experience, together with the Cono Sur Intergovernmental initiative launched by the PAHO in 1991, marked a major advance in the country to eliminate the main vector *Triatoma infestans*, to interrupt transfusional transmissions, and to generate evidence for the 1998–2002 Bolivian Chagas National Program (ChNP) proposal [39, 40]. Since then, the Ministry of Health, the Departmental Health Service (SEDES for its acronym in Spanish), and the National and Departmental Chagas Programs have been working in coordination to control both the vector and transfusional transmissions, and to provide epidemiological surveillance, housing improvements, and community education. Furthermore, in 2006, Chagas disease was recognized as a public health priority by the Bolivian Government (Law No. 3374 of March 23, 2006) [41]. In 2017, the Ministry of Health published the first manuals for the detection and treatment of infant and congenital Chagas disease to prioritize health care for acute and pediatric infections [42–44]. However, the lack of regulation has yielded insufficient results, and Chagas disease remains a public health challenge [45]. The prevalence of *T. cruzi* infection among Bolivia’s population decreased from 20% in the 1980s to 6.1% in 2010, a figure that remains noteworthy [39, 46]. In 2010, new cases of vector-borne transmission and vertical transmissions were 8,087 and 661, respectively [46].

With an unprepared public health structure, NGOs such as *Médecins sans Frontières* /Doctors without Borders supported the implementation of primary health-care programs for Chagas disease in rural districts of the departments of Chuquisaca and Cochabamba, offering assistance to children, adolescents, and adults (1999–2016) [25, 45, 47]. This experience demonstrated the need to assist the adult population, but there were no protocols or guidelines for the management of chronic adult patients at national level as part of the ChNP. Even though a committee of experts developed a comprehensive care manual for adults with Chagas disease in 2011, it has not been published by the Ministry of Health to date. On the other hand, after a pilot program (2004–2010) with the support of Belgian cooperation, a protocol was introduced in 2011 to control the transmission of congenital Chagas and medical care for newborns [48, 49]. In 2009, the Bolivian Chagas Platform was established by the Barcelona Center for International Health Research (ISGlobal)-Hospital Clínic, and the Foundation for Science and Applied Studies for Health Development (CEADES for its acronym in Spanish), together with the ChNP and the support of the Spanish Agency for International Cooperation and Development [36]. The Bolivian Chagas Platform is a vertical care system under the umbrella of the ChNP that has established six specialized centers for the management of chronic Chagas disease in the departments of Cochabamba, Tarija, and Chuquisaca. This collaboration has provided guidelines and protocols, common database, capacity building, and clinical studies [36]. Since then, a strategy of decentralization of Chagas disease health care has been gradually implemented by training health personnel from 50 primary health facilities in 27 municipalities and creating networks that provide Chagas disease health care [36]. These networks in Bolivia are linked to the Hospital Clinic of Barcelona in Spain with mutual learning and shared protocols, allowing a transnational attention to a population that moves, relates, and communicates intensely in both countries [50].

### A bottom-up research: Relationship with the literature

After ten years of health-care provision through the Bolivian Chagas Platform, there are still marked inequalities, vulnerability conditions, and a complex psychosocial dimension that hinders or impedes access to health care for Chagas disease. In this regard, there are major differences between rural and urban environments, genders, and population groups with different socioeconomic conditions [50, 51]. This highlights the need to understand Chagas disease from a social perspective, as well as to establish horizontal dialogues with civil society to generate useful knowledge to improve the living conditions of people who might be affected by or living with Chagas disease.

CEADES initiated a *Science Shop process*, a bottom-up research approach that emerged directly from social demands, recognizes civil society groups as partners in the process, and gives them independent advice based on scientific evidence. This initiative was undertaken within the framework of the EU-funded project Ingenious Science Shops to promote Participatory Innovation, Research, and Equity in Science (InSPIRES; 2017–2021) and the Horizon 2020 program, GA N° 74167. The InSPIRES project aimed at co-designing, implementing, and evaluating innovative models for Science Shops as a tool for reducing the gap between science and society [52–54]. CEADES held a workshop in Punata in October 2017 in which community participants and representatives of the Association of *Corazones Unidos por el Chagas* from Cochabamba (ASCUCHAC) raised concerns that triggered various research projects [55]. The following year, CEADES decided to address other priorities also highlighted at the workshop: living conditions under Chagas disease, family consequences, and health-care demands. (See S1 File).

These needs met the evidence expressed in the literature regarding the importance of further research on contextualized sociocultural factors associated to Chagas disease. Furthermore, they require a qualitative evaluation of policies and programs, the identification of health seeking behaviors, and the design of transnational approaches to understanding the social phenomenon across borders [10]. The social determinants of Chagas disease have been described by various authors in different countries, such as Bolivia, Argentina, Brazil, United States, Spain, and others [3, 10–12, 56–59]. Even though national and local contexts have their own particularities, many of these social determinants are common between endemic and non-endemic countries, and across urban and rural areas [10]. The most pronounced aspects are the psychological impacts of the diagnosis and the widespread sociocultural representations that connect Chagas disease with poverty and, hence, with stigmatization and social exclusion [10–12, 58, 59].

Thus, in October 2018, with the approval of the CEADES Scientific Committee and the revalidation of ASCUCHAC (an essential step of the Science Shop process), a group of researchers formulated the question: *“How do people living with T. cruzi and their families cope with Chagas disease in the Valle Alto of Cochabamba, Bolivia?”* Research teams from CEADES and ISGlobal (partners in the implementation of the Bolivian Chagas Platform and the InSPIRES project) conducted this study together. Afterwards, a reflection on the results of similar transdisciplinary processes in Bolivia and Spain exploring social determinants in the health-care access for Chagas disease will be disseminated under the term Transnational and Transdisciplinary process, a type of collaborative multi-center approach among several countries and promoted by InSPIRES.

The aim of the present study is to explore the social determinants in the living realities of those affected by Chagas disease. Personal and familiar stories highlight factors that generate inequality. Thus, the study provides insights on how families in the Valle Alto of Cochabamba cope with *T. cruzi*/ Chagas disease.

## Methods

### Study design

This is a case study based on life stories immersed in the Science Shop process. By navigating through the research subjects’ stories at different stages of their lives together with them, we discovered and reflected on the realities surrounding Chagas disease, as they are perceived and felt by families in the Valle Alto of Cochabamba.

The results of the study were used to develop a fictional graphic account enriched with testimonies from the three participating families as material for communication and dialogue with the community about what it means to live with Chagas disease and how to advance in the management of the disease. In September 2020, the conclusions and the graphic story “Living with Chagas: Severina’s story” were disseminated in an online event to ASCUCHAC and other actors of the community of Punata to think together about the usability of the findings and materials.

### Selection criteria

Initial research subjects over 18 years old were selected in collaboration with the health personnel of the Bolivian Chagas Platform of Punata through facility-based sampling [60]. Personnel of this health center summoned patients who had undergone the treatment for *T. cruzi* infection during 2018 and invited them to an informative meeting. Two out of three attendees were interested in collaborating, accepted the involvement of their families, and met the following inclusion criteria:

a At least one family member undiagnosed for *T. cruzi* infection and not intended to seek a diagnosis.
b At least one family member had a positive diagnosis of *T. cruzi* infection and did not undergo treatment or medical follow-up.
c At least one family member with *T. cruzi* infection who underwent treatment or medical follow-up.
d At least one mother in the family who had been screened for *T. cruzi* infection during pregnancy.

Willingness to participate in the study was prioritized because of study participation requirements related to time and openness to share their life and family stories. No other family characteristics beyond those above mentioned were considered requirements for participation. According to the objectives, selection criteria, and resources of the study, two families and eight individuals were predicted in the design phase.

Using the snowball sampling technique, other adult family members cohabiting in the same or different household with the abovementioned profiles were also invited to collaborate [60]. The family members involved considered other relatives fulfilling the criteria that may be open to share their lives and talk about Chagas disease. Thus, we interviewed other family members with characteristics that would otherwise be hard to reach, such as individuals who did not access health care despite having information about Chagas disease from other family members [60]. In the first family, six people were considered and four agreed to participate. The household is composed by four adults of which three were interviewed (Severina, Manuel and Janet). Other relative from the extended family and different household also participated (Silvia). In the case of the second family, a couple of two adults who conform the household were interviewed (Victor and Arminda). Although four people were tempted to participate, it was not possible to access other members of the extended family as their commitment to the study faded due to lack of time. Nevertheless, it was decided to include this interview in the analysis because of the richness and relevancy of the testimonies for the research. Consequently, the third family was included to cover all the profiles. Three out of eight people in the third family engaged to the study (Marta, Miguel and Jenny), cohabitants of a household with five adults.

### Data collection and analysis

Narrative research focuses on individual stories across life stages. It offers a better comprehension and theorization of phenomena by digging into real life stories. Thus, it focuses on the conceptual depth of the emerging categories rather than on the saturation point or heterogeneities in meanings [61]. Besides, continuous reflection on the richness and thickness of the data, and therefore the sample size needed, was discussed in the analysis stage. Finally, nine subjects in total were interviewed.

Life stories were collected between November 2018 and March 2019 using in-depth interviews and observations. The In-Depth Interview Guide gathered open-ended questions aimed at raising topics of conversation and collecting testimonies (See the document in S2 File). All interviews were recorded with the approval of the interviewees. In addition, field notes were compiled using observation techniques. In-depth interviews lasted between 42min and 2h20 and were individual in most cases, except when the interviewees preferred the presence of another family member or they joined the conversation punctually. Some In-depth interviews were conducted in Quechua and/or Spanish by a bilingual researcher of the team (Spanish and Quechua), accompanied by two or three observers. See Table 1. Afterwards, information about Chagas disease was provided, and many doubts were resolved. In addition, efforts were made to facilitate access to the Bolivian Chagas Platform for those who showed interest.

**Table 1:** Complementary information about the in-depth interviews undertaken.

	Family 1				Family 2	Family 3		
Interviewees (Fictitious name)	Severina	Manuel	Janet	Silvia	Victor and Arminda	Marta	Miguel	Jenny
**Relation to the first family member intervieweed**	(-)	Severina’s husband (share household)	Severina’s and Manuel’s daughter (share household)	Severina’s sister (different household)	Marital couple (share household)	(-)	Marta’s husband (share household)	Marta’s daughter (share household)
**Sampling**	Hospital-based	Snowball	Snowball	Snowball	Hospital-based	Hospital-based	Snowball	Snowball
**N of interviews**	2	1	1	1	1	1	1	1
**Companions**	Manuel	-	-	-	-	Felicidad (Marta’s sister) and Jenny	-	-
**Interviewer**	N. Mendoza	N. Mendoza	N. Mendoza	N. Mendoza	F. Zapana	F. Zapana	N. Mendoza	N. Mendoza
**Observers**	I. Jimeno & F. Zapana / F. Zapana & C. Billot	F. Zapana & C. Billot	F. Zapana & C. Billot	I. Jimeno & C. Billot	N. Mendoza & C. Billot	N. Mendoza, I. Jimeno & C. Billot	I. Jimeno & C. Billot	I. Jimeno & C. Billot
**Place of interview**	Family home, Cliza municiplity	Family home, Cliza municipality	Family home, Cliza municipality	Family home, Cliza municipality	Family home, Punata municipality	Family home, Punata municipality	Family home, Punata municipality	University campus of Cochabamba
**Date of interview**	17–11–2018 / 20–11–2018	15–12–2018	20–01–2019	25–03–2019	03–02–2019	02–03–2019	18–03–2019	21–03–2019
**Interview duration**	42’’ / 1H20	1H37	1H00	1H30	2H20	2H	50’’	45’’

Recordings were transcribed and those in Quechua were transcribed into Spanish using codes to emphasize modes of expression and non-verbal language. The quotations included in this manuscript were translated into English adding some terms in the original language. Researchers performed a qualitative content analysis that included elements of linguistics to detect common expressions in the Valle Alto region, and also an interpretative analysis giving meaning to what research subjects partially told in their testimonies or what they said without intending to [62]. Prior to fieldwork, researchers defined preliminary themes and categories based on the literature findings and theoretical and empirical contents from the Bolivian Chagas Platform experience (See S3 File). Three major components (structural, psychosocial, and the health system component) conformed our preliminary theoretical framework inspired on the Social Determinants of Health by the WHO and the Multidimensional Framework for Access to Chagas Disease Healthcare by Forsyth C. et al. [37, 63]. This stage served as a common starting point for the study researchers, although inductive analysis prevailed.

Researchers coded individually all data from the narratives of the transcriptions, rescuing the predefined themes but, above all, capturing the themes that emerged from the testimonies, conforming interrelationships among them, and giving interpretative hierarchy meanings. More weight was given to those categories on which research subjects themselves placed the emphasis. Researchers coded and categorized the transcripts and conducted a triangulation process in several rounds of data analysis by contrasting, restaging, and finding intersubjectively validated results (see dataset in S4 File). Triangulation processes allow the use and comparison of multiple data sources, researchers, theories, and methods to increase the reliability of data and its analysis and to give robustness to the conclusions of the study [64]. In addition, they enable a diversity of perspectives on the same phenomenon [64]. The research team brings together experience and expertise in social science research, intercultural health in Bolivia, migration and health, gender and equality, public health and international health. The entire analytical process started from the beginning of data collection and lasted until December 2019.

### Ethical statement

The present study was approved in November 2018 by the Ethics Committee of CEADES (IRB N°0990–0279/FWA:00024189), guaranteeing the relevance and the design of the study in accordance with the principles expressed in the Declaration of Helsinki [65]. Ethical reflections were prominent throughout the project considering that individuals met vulnerability conditions associated with Chagas disease.

Voluntary collaboration was certified by a signed declaration of informed consent that acknowledged the rights of rectification and withdrawal whenever they wished (See S5 File). Sufficient time was devoted to reading and carefully explaining this document to them, as well as to answering all their doubts. The audios of the interviews were recorded with the interviewees’ oral and written consent. Interviewees’ confidentiality was ensured by the anonymization of the data collected in all the reports, assigning a code and a fictitious name to each individual. All the documents and files of the study were stored in a cloud storage platform to which only the research team had access. The original paper documents with the interviewees’ informed consent were stored in a locked cabinet at CEADES headquarters (Rico Toro 1054, Queru Queru, Cochabamba, Bolivia) with sole access to the people responsible for the study. In addition, specific data characteristic of the families that could identify them were eliminated from the dissemination products to avoid stigmatization and discrimination and to preserve fundamental rights.

### Research subjects and study setting

The Inter-Andean valleys of Cochabamba are composed by three municipal associations: Valle Alto, Central Valley, and Cono Sur. The Valle Alto includes 15 municipalities with similar geographic and socioeconomic conditions, characterized by rural districts with a long tradition of agriculture and cattle raising and some urban districts as commercial points. Today, the agricultural tradition continues and is accompanied by other economic activities such as trade, manufacturing, construction, education, and transportation [66]. The ethnocultural social basis in the region has been shaped by Quechua inhabitants, mainly during and after the Colonial period [66]. However, ethnic identities have a fluid and dynamic cultural character, without the possibility of applying the dichotomous indigenous/non-indigenous vision [67]. Language is an element of identity production that enters the ethnicity distinction [68]. Therefore, it was used to describe ethnocultural characteristics.

**Fig 1:**
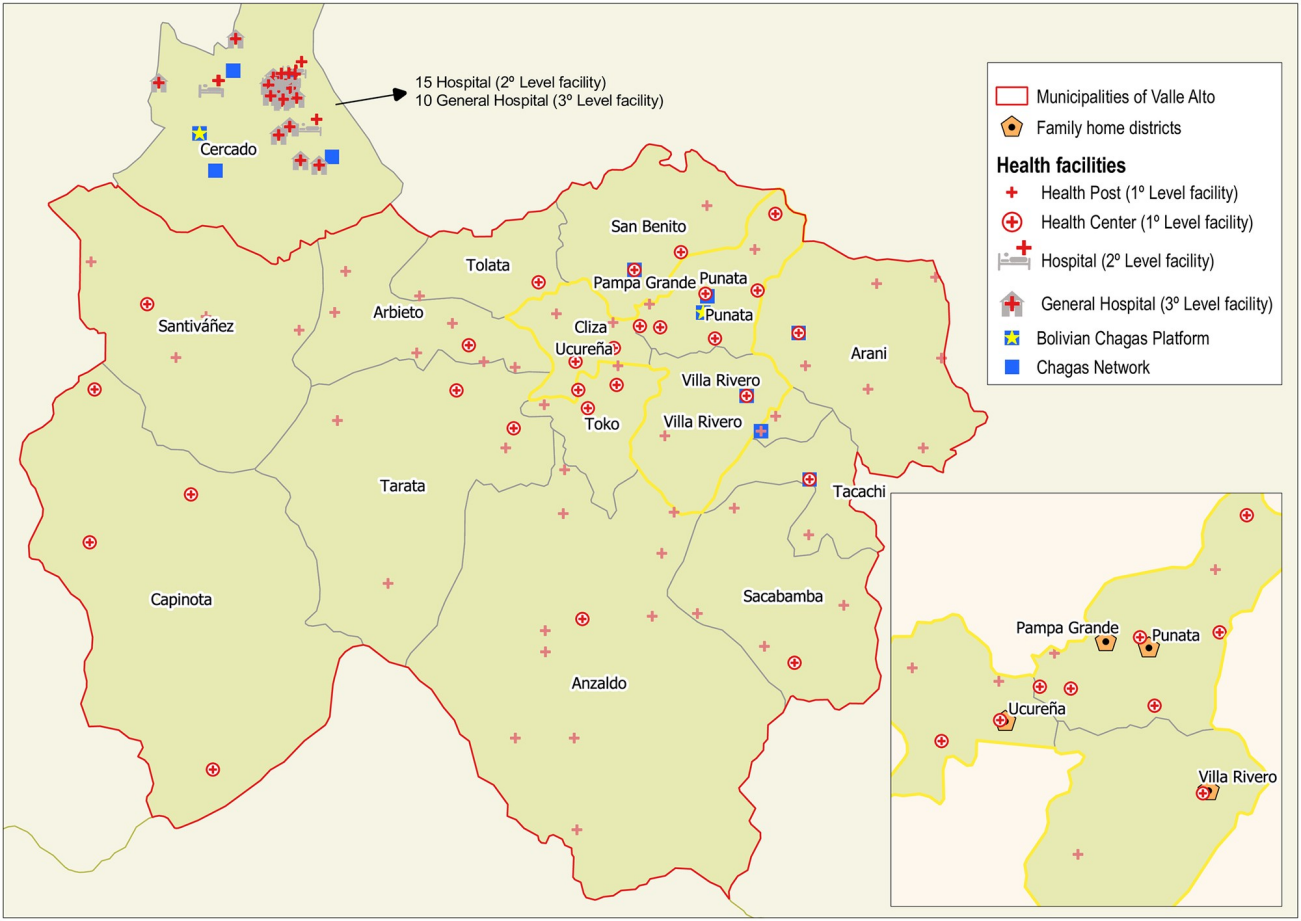
Map of the Valle Alto of Cochabamba and the health facilities in the region. Own creation from cartography of the Ministry of Bolivia 2016. [72]. In addition, it shows the municipality of Cercado where the capital Cochabamba as well as the second- and third-level facilities are located. The Bolivian Chagas Platforms and the Chagas Networks are highlighted. The expanded box shows the districts where families have resided.

The National Health System in Bolivia is organized into the short-term social security, private and public sectors. The social security sector comprises the health funds (*¨Cajas de salud*), university insurance companies, and the General Directorate of Health. The private sector is composed by private for-profit health service providers (private insurance services and clinics), non-profit organizations (non-government organizations and Church) and the traditional medicine subsector represented by the Vice Ministry of Traditional Medicine and Interculturality. The public sector, through the Ministry of Health, establishes four levels of management: national, departmental, municipal and local, and offers the Single Health System (Sistema Unico de Salud—SUS) to people who are not protected by the Short-Term Social Security subsector and to unprotected foreigners whose country of origin does not have reciprocal health agreements. Public health services in Bolivia are distributed into four levels of facilities [69, 70]. First-level facilities provide primary health-care services, health promotion, and disease prevention. In the Valle Alto region, rural districts operate with a health post that offers nursing services and community health promotion. Meanwhile, in urbanized locations there is one health center that provides primary medical care. Second-level facilities include some medical specialties and emergencies. In the Valle Alto region, there is only a second-level facility in Punata, whose hospital is in the process of being upgraded. Third-level facilities comprise all services and specialties, including cardiology and gastroenterology, and are located in the city of Cochabamba in Cercado. Fourth-level facilities refer to specialized medical centers. However, in the department of Cochabamba there are no facilities with these characteristics. Punata is the reference for the Bolivian Chagas Platform in the Valle Alto area. This platform has built a Chagas Network that links first-level health facilities that provide health care for Chagas disease [36] (Fig 1). The Bolivian Chagas Platform calculated a prevalence rate of 21% of *T. cruzi* infection among people assisted in the municipality of Punata in 2017 [71]. Figures are very heterogeneous across districts due to under-reporting and heterogeneous quality of data, with prevalence rates of *T. cruzi* infection reaching up to 60% in some areas [48].

**Fig 2:**
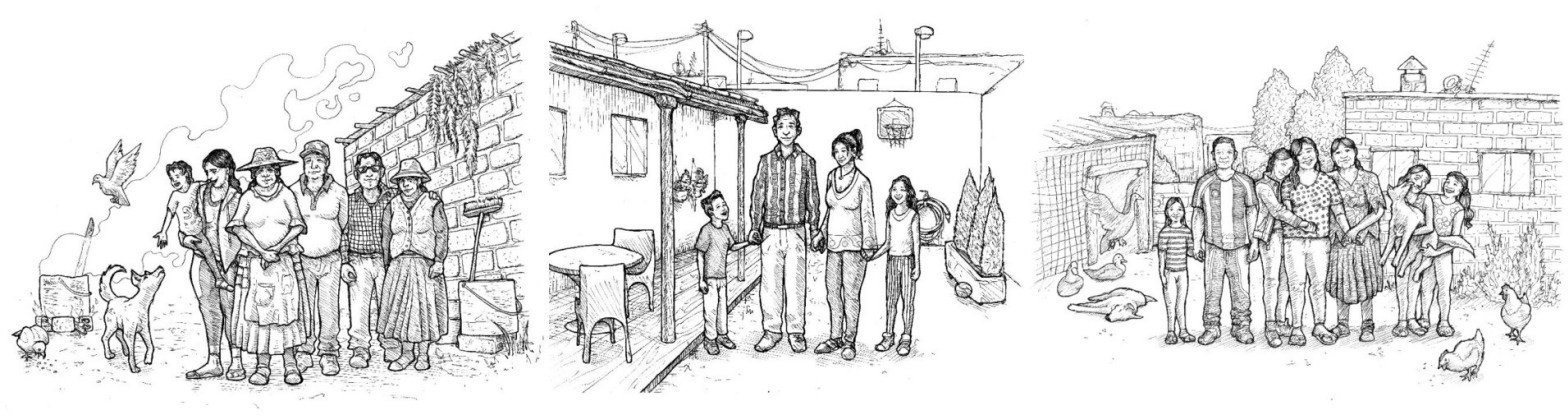
Illustrations representing the most affectively relevant people for each family’s story. Family 1: (on the left) *Severina* with her husband, *Manuel*; their daughter, *Janet*, carrying her own daughter; and Severina’s sister, *Silvia*, with her husband. Family 2: (in the middle) *Victor* and *Arminda* with their children. Family 3: (on the right) *Marta* being hugged by her sister and her older daughter *Jenny*. Next to *Jenny*, Marta’s husband, *Miguel*; their three other daughters.

The families in the study resided in rural and periurban districts of Punata (Punata, Villa Rivero and Pampa Grande) and Cliza (Ucureña) (Fig 1). Their family structure has the typical characteristics of the Valle Alto. In socioeconomic terms, they present a medium socioeconomic status, which is common in urban areas of the province, characterized by a diversified income through different occupations. Although agriculture is not the basic means of subsistence for these families today, they continue working on their parents and grandparents’ lands. Table 2 summarizes the main descriptive characteristics of the study group. In addition, Fig 2 presents fictitious illustrations.

**Table 2:** Subjects in the study grouped by families and their main general characteristics.

	Family 1				Family 2		Family 3		
Ficticious name	Severina	Manuel	Janet	Silvia	Victor	Arminda	Marta	Miguel	Jenny
**Gender**	woman	man	woman	woman	man	woman	woman	man	woman
**Age**	57	59	30	55	38	37	45	41	19
**Level of schooling**	Secondary	Higher education	Higher education	Higher education	Secondary	Secondary	Primary	Higher education	University education
**Occupation**	Farmer Housewife	Rural teacher Farmer	Rural teacher	Rural teacher	Merchant Musician Builder	Housewife	Merchant Artisan Housewife	Construction Technician	University student
**Language preference**	Mostly Quechua	Quechua and Spanish	Mostly Spanish	Mostly Spanish	Mostly Spanish	Mostly Spanish	Mostly Quechua	Quechua and Spanish	Mostly Spanish
**Diagnosis of *T. cruzi***	Yes (+)	Yes (+)	Yes (-)	No	Yes (+)	Yes (+)	Yes (+)	Yes (+)	Yes (+)
**Treatment and/or follow-up**	Yes	No	(-)	No	Yes	Yes	Yes	No	Yes
**Screening during pregnancy**	No	(-)	Yes	No	(-)	Yes	Yes	(-)	(-)

## Results

**Fig 3:**
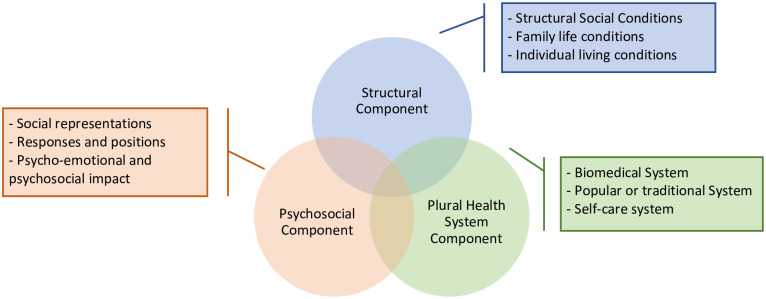
Emerging theoretical framework.

Our findings structure and theorize over emerging categories from the testimonies (Fig 3). Although divided into components for expositional purposes, all categories are closely interrelated with each other.

A **The structural component**. It refers to structural factors that determine exposure to Chagas disease and influence social constructions and means of understanding the disease and health care:
  (a) *Structural social conditions*: macrosocial factors such as the sources of economic and political development and the social and cultural context of the Valle Alto of Cochabamba and Bolivia.
  (b) *Family life conditions*: family composition and structure, socioeconomic level, material circumstances, family resources, housing, and exposure to triatomines.
  (c) *Individual living conditions*: gender, age, ethnocultural group, education, and occupation. (Table 1).
B **The psychosocial component**. It describes the different ways of understanding, living, and communicating health-care experiences. The starting points are the collective subjectivity in the understanding of health and disease and the acknowledgment that the strategies to treat a condition, live together, solve, or eradicate the disease are diverse:
  (a) *Social representations of Chagas disease*: cognitive elements, stereotypes, opinions, beliefs, values, and norms socially elaborated from experiences, information, or thought models that are received and transmitted [73].
  (b) *Responses and positions toward Chagas disease*: social patterns in vector control, the search for diagnosis and medical care, and commitment to care and restore individual and family health.
  (c) *Psychoemotional and psychosocial impact on the lives of affected families*: impact on the personal, family, social and occupational spheres. In addition, this section contains relevant issues such as stigma and discrimination as well as processes of inclusion and social support.
C **Plural health system component**. It refers to the knowledge, practice, and pluralistic relationships between people and health systems. Health-disease-care processes are woven around the different health-care models that overlap and complement each other [74]:
  (a) *Biomedical system*: public health establishments and private clinic institutions.
  (b) *Popular or traditional system*: health care for the diagnosis, prevention, and suppression of physical, mental, and social disorders made by to traditional therapists such as healers, witch doctors, shamans, etc.
  (c) *Self-care system*: individuals diagnosing, explaining, attending, and relieving themselves in an autonomous manner.

Results are found in several chapters that explain the complex relationships among these three different components. This manuscript focuses on the main social determinants of each component that interferes with the access to health-care for Chagas disease in the context of the Valle Alto of Cochabamba (A. Structural component, B.a Social representations of Chagas disease and C. Plural health system component). In-depth results about the impact of Chagas disease on family life (B.b Responses and positions toward Chagas disease & B.c. Psychoemotional and psychosocial impact on the lives of affected families) will be presented and further developed in other two scientific publications (Forthcoming).

### A. The structural component

#### A.a and A.b Social and family living conditions

Research subjects identified structural factors that determine the exposure to infection, the development of the illness, and the access to health care. However, their perceptions on the causes of Chagas disease have changed throughout their life history and have been affected by urbanization and progress in social and economic status. In this region, environmental characteristics and living conditions favor the intra- and peridomestic colonization of *T. infestans*, known in the area as *“vinchuca”*. It frequently occurs in natural constructions that deteriorate more easily and where vinchucas find a suitable habitat. However, adobe houses are locally owned constructions that are environmentally responsible, bioclimatic, and economical. Therefore, the vector problem lies in the precariousness, lack of knowledge, or insecticide resources rather than in the construction material. Research subjects lived with their extended families in these circumstances in their childhood, and even continued to do so in adulthood. According to research subjects, vinchucas were part of the ecosystem, so for a long time their presence was normalized and naturalized. Recognition of the danger of vinchuca was installed with vector control and housing improvement programs in which families were involved and committed. Knowing that vinchuca transmits Chagas disease, cohabiting with the insect became frustrating and even terrorizing due to the high rate of infestation and reinfection.

*“The first impression when seeing these types of animals, not knowing the information, well … I took it as any type of animal, or any other type of bug, like a fly or other type of insect. And I didn’t know anything else” (Janet, family 1)*

*“What am I gonna say*…, llakiy, *(suffering), there was no help, neither from the government nor from the authorities. Later they came, as I say, those gentlemen to fumigate. I said, ‘Thank you’ … And vinchucas finally disappeared. There were a lot: on the sheep, on the pigeon, on the cow, on the pig, everywhere! I remember it and it’s incredibly sad. I’d have always taken photos, but there was no time to take photos, nor was there a cell phone…. We didn’t want to get sick, we wanted to live and because we wanted to live, that is why we worried. We revoked the house.” (Severina, family 1)*.

According to the stories, once the battle against vinchuca was won, the feeling of the risk of Chagas disease disappeared and became a problem of their past. Although they already knew that they might have a disease because they were in contact with the vector, not having much information about the symptoms, how the disease develops, treatment, and where to seek care, made them forget it or live in relative uncertainty and calm. Today, exposition and suffering from Chagas disease is related to marginal and rural communities in a situation of poverty, with scarce resources and many needs, where vector control actions did not reach them or were not maintained. Diverse social structural problems arise in these contexts, and Chagas disease is only one of all the concerns. It also stands out how it affects migration abroad, leaving empty houses, places where reinfections occur and become an infectious focus for the rest of the community.

*“As a rural teacher, I’ve traveled almost everywhere in Cochabamba. Once, there was a mishap, and we were halfway there. A local person lent me a* ‘payasita’ *(straw mattress) and a sheet. They took me to the yard! I was really surprised; this family doesn’t have a cot! I went to sleep, and I woke up and heard a little sound. I was a little scared. “Where’s that coming from? The rain is coming, or is it the wind coming?” I took my sheet off, I looked around… when… The vinchucas! They were coming toward me! As ants go. (…). So that sector is an endemic place. (…) What I recommend is that doctors visit those families in need, so that they can help them. Because in those places where I’ve gone to work, families are extremely poor. If families get attention, it’s within a specific campaign for a certain time until the project is finished. The project is over, just done there. So doctors do not follow-up.” (Manuel, family 1)*.

Currently, admitting the presence of triatomines in their lives seemed to be embarrassing. The adobe house has become stigmatized because of the direct relationships with the vinchuca, rurality, and poverty. Otherwise, adobe houses and coexistence with vinchuchas were the main elements that led families to suspect that they might have Chagas disease, since other forms of transmission are not very well known. Having a relative affected or dead by the disease is another of the crucial determining factors of the risk perception.

*“Most of all, there are neighbors, relatives, and my wife’s relatives… In their house too (there is vinchuca), worse than in my house. Over the years, this is how they were dying, dying, like this. But, young people, young people! Then, when I heard that they had died—‘it’s said that they had died: They surely died with Chagas.’ I remembered. Me too. Is it that I’m running the same fate of them?” (Manuel, family 1)*.

Certain occupations might result in greater exposure to infection. Members of families 2 and 3 claimed to have seen vinchuca in rural communities in their service as teachers or builders. Although teachers received specific education about Chagas disease and even participated in rapid test campaigns, the information received focuses on vector control. Thus, it can be observed among the families interviewed that the level of schooling did not provide more updated scientific knowledge of the disease and greater access to health care for it.

#### A.c Gender issues in health care for Chagas disease

This study includes one specific female profile in the selection criteria to measure how a positive diagnosis during the systematic detection of Chagas in pregnant women affects wellness and mental health. According to some women who participated in the study, hospitals did not perform these tests, or the results were not reported. On the other hand, one of the research subjects, *Marta*, did not attach any importance to the positive diagnosis or continue follow-up care for her newborn. It took her another eight years to reach biomedical health care for Chagas disease for her and her daughters. In this regard, the procedures and messages of the health system do not seem adequate or effective.

*“I was pregnant with my youngest daughter; I went for a check-up, and they told me that I’d Chagas disease. They said, ‘You’re going to get treatment when your daughter is born.’ But I didn’t believe them either. ‘It must be a minor illness,’ I thought. So, I didn’t do it. (…) They told me that I had to take a lot of tablets and that they were extraordinarily strong. So that must be (the reason why I didn’t undergo the treatment), or I don’t know why. (…) (When the girl was born) the doctor told me, ‘You will bring her back in a month.’ But I didn’t take her because she didn’t get sick.” (Marta, family 3)*

*“Since (my mother has Chagas), there’s a chance that I have it, and maybe my baby can have it, too. Then, I remembered that when she was born, they did tests and didn’t tell me anything. They tested her, and the test result was negative. Then, they didn’t test me… no, they didn’t. They just gave me the treatment for premature birth, nothing else. Then, the baby had all sorts of tests done and nothing. (…) (And did they do it only once at birth?) Yes, nothing else. (…) As I’m nationally insured at the Seton, at the Petroleum Health Insurance… The teachers’ union belongs there. All the time I was pregnant I wanted to be treated there. Apart from that, I had a private gynecologist. The difference is quite big, so I preferred private assistance.” (Janet, family 1)*

According to informants, although both genders, men and women, generally contribute to the family economy, the responsibilities of household chores and care of children and other family members are traditionally delegated to women. Meanwhile, from the interviews performed it emerges that the role of men is to provide and satisfy material needs; they have the responsibility to guarantee the family’s wellbeing. Gender roles may influence decisions in the search for health care for Chagas disease. Regarding the CEADES experience in the Bolivian Chagas Platform, most of the attended people are women. Families 1 and 3 expressed their reasons for seeking (or not) health care for Chagas disease. In the case of the women interviewed, the main motivation was to stay alive to care for their children and family. These women also play a significant role in informing and accompanying other family members and peers for diagnosis and health care. In the case of male subjects, potential labor and social discrimination associated with the diagnosis of Chagas led them to rule out the possibility of seeking treatment.

On the other hand, one of the facts that emerged as very worrisome for a research subject, Severina, was that her daughters were respected and not mistreated by their husbands. Gender-based violence is supported on power inequalities between men and women. Furthermore, men may overcome women’s subordinate opinion in a discrepancy over how to act against Chagas disease. Marta acted without her husband’s permission, since he opposed to treatment. Unfortunately, she ceased treatment due to serious ADRs, which reinforced the position held by her husband and other family members.

*“My wife told me, ‘You can also do treatment’, and I told her, ‘to do that, you have to do an analysis of everything!’ It wasn’t something to do just like that. It isn’t a joke! And that is why she was sick. (Did you accompany her when she went to the Platform for diagnosis?) No, she went there without my permission. Her sister took her!” (Miguel, family 3)*.

### B. The psychosocial component

#### B.a Social representations: Between an inadvertent disease and a fatal disease

Chagas disease is silent, with an asymptomatic stage, in clinical terms, that can last a lifetime or take 20–30 years for symptoms to appear. However, the very name “Chagas”, or “Chagas disease”, is commonly used to refer to both *T. cruzi* infection and the symptomatic condition of Chagas disease in both biomedical and community settings. However, the conception of disease and illness (enfermedad, in Spanish) is commonly associated with a condition or symptoms that prevent people from eating, moving around, and performing daily activities on their own. Thus, a person who does not suffer, or who does not feel sick, will hardly perceive the need to seek specific attention. After 20 years of living with the vector and the following 36 years of being aware of Chagas disease, one of the research subjects, *Silvia*, prefers to remain uncertain due to the lack of symptoms and the prioritization of other occupations.

*“When we feel sick, we always go (to the medical center of her insurance provider). Feeling fine we don’t go easily. (‥) That time my husband didn’t go for tests, until he felt badly. Then they detected that he had (Chagas). My husband recommended that I should go later. (…) At that time my husband was going; as my sister felt sick, so I told her ‘Go, you have time, go with him.’ (…) And I did not go! At that time, it seems that I didn’t have time. (…) Well, I want to be diagnosed. (…) Because my sister has it, that is why maybe I have it, too. Because we have been with a family in a house where we had lived. (…) But no, I cannot.” (Silvia, family 1)*.

On the other hand, research subjects understand the asymptomatic and symptomatic phases of the disease as Chagas (the parasite) remaining in the body without causing harm when its “sleeps” (*Chagas dormido*) until it “wakes up” and causes health problems (*Chagas despierto*). Likewise, there is the notion that the treatment is overly toxic, compromising the body’s resistance. Some subjects think that treatment is precisely the trigger that provokes the awakening of Chagas pathology. These explanations are very much in line with the scientific knowledge from the old paradigm in which there were doubts about the mechanism of immunity and the etiological treatment. However, despite the scientific advances in these areas, this notion is mostly established among people from all sectors of society, including the health personnel of the Valle Alto region.

Thus, several beliefs and positions arise from these constructions. *Not believing in Chagas*, is mostly associated with the belief that it only affects weak and elderly people with previous illnesses so that the cause of death is not properly due to Chagas disease. In contrast, there is also the perception that *Chagas is a psychological illness*, and the concern of receiving a positive diagnosis or even thinking and talking about it, may awaken Chagas. In both positions, Chagas is preferably made invisible, evaded, and denied. In the words of some of our research subjects, when it comes to Chagas, there is no need to rummage around (*No hay que hurgar*).

*“I have Chagas disease. They (the relatives) told me: ‘What have you done the treatment for? Because at your age it is no longer useful! You have gone in vain! Chagas is like that. When the doctor does not look at (attend) you, it is asleep. And when the doctor looks at it (attend the disease), it gets up. What have you gone for? We do not believe in that (Chagas disease).’ (…) They say that although they lived with (vinchuca) …, ‘That is in vain, you should not get rummaged with the doctor. What’s more, it (Chagas) is denied.’ (…) I have been scolded for that: ‘There is nothing to do’, they have told me. (…) (And what do you think?) I think it may be true.” (Marta, family 3)*.

Even though the three families had been committed to vector control programs, they did not perceive the need to seek medical attention for a long time. According to interviewees, the search for diagnosis and health care arises from suffering from some symptoms suspected of being Chagas or from the warning of a family member who has already been attended. Interviewees characterize Chagas as a disease that starts with diverse symptoms such as dizziness, chest pains, swelling, and exhaustion. The expectation of death stigmatizes Chagas disease, and it is better to avoid communication in the social environment in view of the isolation, discrimination, and social and labor exclusion. Besides, because of the psychoemotional impact on their families, Chagas disease remains a family matter, while ultimately the decision of undergoing diagnosis and treatment is personal.

*“I got tested at my educational unit, with the puncture, with the gout. There, the doctor from the laboratory did not give us the results, but the director of the educational unit is the one who communicated them to us: ‘Yours is positive, you have to do treatment’, she said in secret. So, I thought ‘No, no, I do not want to listen, I do not want to listen anymore’ That was my criterion. ‘I do not want to listen anymore!’ (…) We talked about it among colleagues, but the result was hidden. Because… What can you think about the colleague at work…? Considering how the problem of Chagas is, its consequences, and then, where we come from… and how we die! (…) That (rapid diagnosis) happened about fifteen or seventeen years ago. (…) So that’s the idea. Chagas problem is sometimes psychological, so when we think a lot, we worry a lot, then this Chagas problem advances faster. It’s preferable… well, ‘We will forget’. Also, among colleagues, ‘We will forget’. (…) Exactly, (Seeking diagnosis and treatment) is a personal decision” (Manuel, family 1)*.

A research subject, *Manuel*, despite having obtained a positive rapid test in 2003 and knowing about the disease for 36 years, decided to remain in doubt to avoid the psychological effect and protect himself. Thus, some of the interviewees revealed doubts and fears of facing a diagnosis that could jeopardize their lives, even knowing the existence of a center specialized in Chagas disease with free assistance. However, others, after a period of postponement, decided to seek biomedical health care thanks to family support and knowledge of proximity cases that undertook the etiological treatment and improved their health.

### C. Plural health system component

#### C.a, C.b and C.c Pluralism in health systems

The choice of attending the biomedical (public or private), the traditional/popular or self-care systems depends on the identification of the type of disease and the accessibility and solutions that each service can offer at each time. Some of the patients have experienced long pilgrimages with successive comings and goings between private clinics, public health facilities, and traditional or self-care practices. Research subjects explained that they generally care for themselves using medicinal plants, based upon the knowledge shared by family members. In addition, they revealed that they occasionally obtained drugs from pharmacies to alleviate an affliction or treat a self-diagnosed problem.

*“(Do you usually go to the doctor?) Almost never. I don’t know, because we’re also insured. No, we don’t try. (…) Because of the time … At home, you must work, do a lot of things … (When something hurts, do you go?) We buy pills from the pharmacy to calm down. And I say, -‘Oh, if I go to the doctor, he will give me injections.’ ‘Do this, then do that.’ … I’d better go to the pharmacy. (…) (At the medical center) you’ve to queue, you’ve to wait your turn, and we lose a whole day. So, I go to the pharmacy, what we get we swallow and that’s it.” (Severina, family 1)*.

A research subject, *Marta*, after stopping the treatment for *T. cruzi* due to severe ADRs for which she had to be hospitalized, was blamed by her relatives-in-law for seeking treatment at the hospital. Instead, they suggested that she take some medicinal plants that they had brought from the Chapare region (a pre-Amazonian basin region in the department of Cochabamba) and that they claimed to be effective against Chagas disease. *Jenny*, her daughter, explained the regular use of medicinal plants in her family to treat diseases.

*Others (relatives) have told me ‘What have you done! You have to take some flowers from* sunchu *(instead of an etiological treatment)!’ (‥) ‘You just have to take those little yellow flowers’, they told me. (‥) Because they brought me some flowers from the Chapare to take for Chagas care. My nephew’s mother-in-law brought me an infusion from those little flowers I had to drink every day, in the morning, all day long.—’That is good because I brought them for many people, and now they are better.’ She told me. (…) I took them after (the etiological treatment) because I told them, ‘That treatment has made me sick. They had me hospitalized’.” (Marta, family 3)*.

*“I’m well used to medicinal plants. (…) I took what my grandmother gave me to heal and that is it. Yes (they cured me) (…) There are many medicinal plants that do help a lot. Unlike when you get tablets and tablets … That doesn’t help you. (…) Tablets, as they say, are a kind of drug. So much to consume. On the other hand, natural remedies don’t affect you at all. They help you to improve your health (…) Chamomile, aloe, güira güira, small yellow flowers, and that mixture is boiled (…) When one has a stroke, for that disease, they use ruda. (…) My aunt now has all the medicinal plants at home! We go there and she gives us the plants.” (Jenny, family 3)*.

Other notions and practices from the popular or traditional system were found related to the magic-religious sphere. The notion of bewitchment (embrujo or hechizo) has been present in the three families as an explanation of a health and social problem difficult to cope with. Severina (Family 1) reported that her aunts were bewitched (embrujadas, in Spanish) and suffered from black and thickened bellies and finally death. In fact, the diagnosis was a form of “bewitchment in vein” (embrujo en vena) as many of the family members met the same fate. Before her death, one of them was diagnosed with Chagas disease by a physician. Miguel (Family 3) states that many of his family members and neighbors thought that his mother was bewitched (hechizada, in Spanish) and should be treated by healers (curanderos). Despite having received a diagnosed with Chagas disease by a medical doctor, when she was informed of the non-existence of a biomedical treatment to cure her illness, she clung even more to the explanation of traditional medicine. Her symptoms were weakness and shortness of breath. For both Severina and Miguel, consequences of embrujo and manifestations of Chagas disease are two ways of understanding the health problem from two different worldviews. Research subjects did not deepen their understanding of the symbolism of bewitchment and the rituals or therapies offered by traditional therapists. It appears that they were not very attached to these beliefs or did not want to be judged by interviewers from the biomedical world. However, *Severina* recognized that the therapist who attended her aunts was able to tell them, in other words, an accurate prognosis. However, in these cases, traditional therapists did not succeed in restoring their health nor did medicinal plants reverse the progression of the disease. Unrelated to Chagas disease, bewitchment also appeared among the second family’s testimonies. Victor recalls that his father has suffered from alcoholism and was initiated, according to her mother, when some relative, out of envy, gave him something to cause harm to the family.

*“My aunts, who have died … had swollen stomachs with this disease. Now we know that it was Chagas; but, at that time, they told us: ‘They have bewitched them (les han embrujado), that is why they have died.’ (…) They went (to the healers), but they didn’t say, ‘I have Chagas.’ They have been bewitched (Embrujadas están)‥” They are bewitched in the vein (Están embrujados en vena), in the blood. The whole family is going to die’, they said. Their bellies became large and black. Currently, they just know that they had Chagas disease. My sister is her goddaughter; she had taken her to the doctor. And the doctor said, ‘No, your aunt is not bewitched (embrujada), she has Chagas disease.’ And because of that, we say that this family is dying of Chagas.” (Severina, family 1)*

*“When I took my mother to the medical doctor; he told us: ‘She will no longer have a 100% cure, no longer’. Chagas disease was what mostly attacked her. (…). Before, most people talked a lot about brujería, o sea, le han hechizado (bewitchment, I mean, she was bewitched). Like something that stays or gets lost. Yes (we have gone to have her treated). More than anything else, that’s what (the bewitchment, hechizo) they thought about…. When a neighbor came in, she said: ‘kayta ruanayki! Kayta ruanayki!’ (in Quechua, ‘do this and do that’). And my mother held on to that. (researcher asked) Did they go to the curanderos (healers)? Yes, they took her to La Paz. My brothers have spent money (on healers), more than anything else! They spent almost three thousand dollars! They have sold one, two, three, almost four family lands. To heal. I said to my brother, “We shouldn’t trust the curanderos (healers) so much, they just take money from us! And that’s why I don’t believe anymore! Because, if she was really bad, it was better to do the treatment and get well!” (Miguel, family 3)*.

*In the place where we lived, my dad was considered, as they say in Quechua, khaparuna (millionaire, rich). And supposedly, my mom tells me that, at that time, his own comadres (godmothers), gave my dad something so that he would become like that… vicious of drinking… that he would become an alcoholic. From that day on, my dad became like that until ten years ago. (Victor, family 2)*

In our study, we found within the same collaborating families, people who spoke Quechua and others, usually the next generation, who knew Quechua and followed the same cultural patterns but preferably spoke Spanish. Transition to Spanish reflects a process of change in socioeconomic status. Intra-ethnic relations between people in different positions of power often highlight differential, prejudiced, and discriminatory attitudes. Research subjects experienced these attitudes in their daily lives, but they were also most noticeable among health personnel in public hospitals.

*“How can I tell you? I don’t like that… Even if we are small, black, or Bolivian or Chilean or Paraguayan… wherever we are from, we are human, we are people, don’t you see? We have the same nose, eyes, mouth… everything we have. So, I cannot discriminate, I cannot differentiate between other people. ‘Not you, but you can.’ Moreover, the most indignant thing is how they (health personnel) treat old people. That makes me even more indignant. I don’t like it.” (Victor, family 2)*

*“The quality of the health system is (regular)* bien no más. *Some attend well, others don’t have patience with us, they don’t explain. What do you feel, this, this… ‘Okay this you will not eat, this you will take, this and this and that.’ They don’t tell you, ‘this is attacking you, so you must take care of yourself; this is a disease that attacks such a thing.’ I’d like the doctors to explain to me more, ‘That’s the beginning of the disease, it attacks this, you have this, that’s why you feel like this, this is how you should take care of yourself.…’ That, I’d like to be told. But they do not. I don’t know why, is it because we do not pay? I say yes.” (Severina, family 1)*

#### C.c Accessibility to the public health system

Interviewees considered that the quality of the public health system is deficient, which discourages them from seeking health services. The three families indicated their preference for private clinics for their health care despite the costs and explained their choice by pointing out some shortcomings of the public system: 1. limited decentralization of services and the consequent geographic and economic barriers, 2. high expenditures needed for attention and medical treatments without guarantee, 3. long-time queues and waiting time that can only be overcome in cases of serious illnesses, emergencies, or hospitalization, 4. administrative barriers and mismatches between levels of health-care facilities and specialties, 5. lack of resources and qualified personnel, and 6. inappropriate and discriminatory attendance. Thus, confidence in the public health system is extremely low. Moreover, they perceive attending private clinics as a quality-assured investment for better health care. However, they explained that many other families cannot afford private or even public assistance because of the associated direct and indirect costs. *Miguel* was tested positive with Chagas disease at a private clinic where he sought health care for another illness in 2017 and did not seek health care for Chagas disease, being in doubt due to fear and myths. In *Victor’s* case, who experienced symptoms, he went to a private clinic to verify his diagnosis, after catching it 16 years before in Argentina. In both cases, the private clinics informed them about the Bolivian Chagas Platform for treatment. The drug for Chagas treatment is purchased institutionally by the NChP to the laboratory producers, and it is dispensed in some public health facilities under strict medical supervision.

*“When I got sick, I went to Cochabamba to a private hospital because I really felt bad. And the doctor told me that I had typhoid fever. I thought it was something else! I had a complete medical examination. The doctor thought I had diabetes, and I said, ‘Look, I want to get everything tested’, and I had everything checked: the heart, the liver… everything. And that’s when the result came out. And the doctor said, ‘You have typhoid fever, and you also have Chagas’.” (Miguel, family 3; He refused to do the treatment)*.

*“Everything the doctor told him could happen. It may affect his heart… Then I told him, “Take care of yourself,” but he never listened to me. He said that he had to work, that he would not have time… ‘But, come on! Take a day, have the complete medical exams done.’ But he didn’t listen to me at all. (Arminda, family 2, talking about her husband) I decided to take the test because I understood that when you have a doubt… They told me that I had Chagas but I really didn’t know if it was real or not. (…) So I lived with that thought (…) Then, I did that study to get out of my doubts. (Victor, family 2) We did it in a private laboratory. At the hospital, he was supposed to make an appointment and wait (Arminda, family 2) ‘Yes, you have Chagas’, said the doctor. And the doctor told me about the existence of the Chagas Platform, a hospital program where I could do the treatment for free. (Victor, familiy 2) He finished the treatment a week ago. (Arminda, family 2)*

One of the research subjects, *Manuel*, through his experience as a rural teacher, was aware of the situation of access to health care in the most remote communities. These population groups cannot afford the economic costs of leaving work, travel, and accommodation to seek medical care. In addition, there are certain beliefs among these people who distrust hospitals and identify them with organ trafficking or categorize them as morgues, where people go to die.

*One problem is the lack of (geographical) access, but also the insufficient economic resources. It is not possible for them to reach. So, well, they subsist as they can. There’s no other way than to bring doctors closer to the communities because they cannot get out. We know very well that a clinical consultation costs fifty dollars and more! So, that is why these poor families do not come here to the medical centers. (…) Transport is now available everywhere; it has improved a lot. It is definitely the economic factor. (…) Well, there is always (discrimination by health personnel against people from the communities). Today, we are trying to make discrimination disappear. But there is always. It still exists in hospitals; well, even in the families themselves! (…) Also because they are not used to big hospitals. They are even afraid to enter hospitals. Fear for the sayings about human and organ trafficking. They hear that it is not safe and that sometimes people die in that place. This creates distrust. (…) Here, in my sector, where I live, I have heard it.” (Manuel, family 1)*

*Marta* presented serious ADRs to the treatment and had to be hospitalized. Nevertheless, *Marta* is determined to try the treatment again despite the messages she received from her husband, relatives-in-law, and the hospital emergency department staff. This confirms that the biomedical sphere is also immersed in the social constructions of the local context. The fees for medical hospitalization were a major economic cost that became one more argument for *Marta’s* husband to oppose the treatment.

*“When I was taking the tablets, I was better. Then I became allergic. Now my heart is beating again, it hurts. I think those tablets were good for me, but my body didn’t resist. I didn’t resist. (…) I was hospitalized for almost two or three weeks. All the time with serums, injections… (…) In the hospital itself, they scolded me. A doctor told me, ‘What have you done this treatment for? You are already 40 years old. Why have you done this to yourself?’ And I said, ‘But others were taking the treatment. It didn’t hurt my sister.’ The doctor said, ‘Others are stronger, you are not strong. The drugs are strong,’ (…) I said I was going to die with my own hands. I did not even feel like getting up. Everything was like that, I was dead. Later, the doctor (of the Chagas Platform told me). ‘You will take care of yourself. If you want, you can come for medical controls, but your husband does not want you to.’ I do want to do the treatment; I will go again. I feel bad now, when I was taking the treatment, I was always fine. I felt better.” (Marta, family 3)*.

Incomplete treatments due to ADRs, ineligibility of etiological treatment for patients with specific health problems or advanced age, and other failures during biomedical health care attendance can lead to lack of adherence in the follow-up of the disease, failure to restore health, and reestablishment of fears and myths. During this study, there was a lack of the national stock of Benznidazole that lasted at least through 2019, and numerous treatments had to be postponed, compromising the trust and credibility of the health system. Study results suggest that there is room for improvement in political commitment and coordination among the Ministry of Health, SEDES, the NChP, the Departmental program and municipal authorities to provide essential medicines for the timely management of Chagas disease.

In contrast, first-level actions such as home visits by health brigades (health teams that visit homes in the community with information, communication, and education activities for health promotion and disease prevention), vector control actions, and educational campaigns in primary schools were very well received by the subjects. An interviewee highlighted how the health brigade helped to raise awareness of Chagas disease and to identify the need for testing. However, she postponed the decision until her sister insisted and accompanied her. On the other hand, school campaigns allow young people to learn about Chagas disease and become familiar with it. However, *Jenny* did not believe the disease was important and took six years to get tested only after a doctor’s recommendation to her mother. The same happened to *Janet*, who received information during her training as a teacher, but spent 18 more years to obtain her negative diagnosis because of the unawareness about treatment.

*“And here some nurses came once to explain about Chagas disease, ‘That’s how Chagas disease makes us feel nauseous, headache, our heart hurts, our stomach hurts.’ At that moment, I decided to go, that is when I said.” (Marta, family 3)*.

*“Yes, (at school) they talked to us (about Chagas), ‘If you see vinchucas take them for analysis. (The vinchucas) bite and when they bite….’ They explained it to you like that. That was almost nine years ago. (…) For me, it was not a dangerous disease before until I saw mom. Then I realized that it is dangerous if you don’t do your test and your treatment in that same time. Only then did I realize that it affects your heart. Before, it was like any little bug, was not it? It itches … it goes away … like this. (…) (And why did you want to do the test after all those years?) The doctor told my mom, ‘You have to bring your daughters to get them tested’. (And what did you think when he confirmed the positive diagnosis?) Easy! Calm down! Nothing scares me! (…) The doctor told me, ‘Right now when you do the analysis as a young person you avoid (the disease).’ If I were an old woman, it would affect me. (…)” (Jenny, family 3)*.

Families 1 and 3 did not know of the existence of the Bolivian Chagas Platform until a family member who had already been treated recommended it to them. On the other hand, health care at this center was well valued. Treated individuals felt well cared and provided emotional calm to their family when they received a negative diagnosis, and after etiological treatment. Patients’ satisfaction is also reflected in the commitment to inform and support other family members. *Severina* became aware of Chagas disease in her twenties and, after doubts and fears, it took another 36 years to reach biomedical treatment thanks to her family’s support. She was referred by her brother-in-law, who had already undergone the treatment and had come on the recommendation of his sisters. *Severina* also took her children to be tested. In Family 3, *Marta* went to the Bolivian Chagas Platform thanks to her sister, who was being treated at the same center on the recommendation of her husband, who is living in Barcelona, Spain. Probably thanks to the transnational approach between the Bolivian Chagas Platform and Hospital Clinic in Barcelona. *Marta* also took her daughters to be tested.

*“My brother-in-law was going to the Chagas Platform and he said, ‘Come on, I’m going there; Doña Severina, you might have Chagas. It would be good for you to know.’ I didn’t dare either. But my sister insisted, ‘You do go! … So, you would know (if you have Chagas)!’ So, I told her, ‘No, the doctors, knowing they always do this and then.…’ But my children told me, ‘I don’t think so. I think it is good to go to be tested.’ My husband also told me that. (…) I said, ‘The doctor is going to tell me that I have the illness, and if he tells me that, I will think about when I’m gonna die. No, I’m not going!’ And my brother in-law said, ‘No, come on, my sisters have also been cured, they have already done treatment, come on!’ And my children also said, ‘Run, go mommy!’ They took me in their arms, and they put me in their own car. (…) Yes, the attention is particularly good. At each check-up, the doctor explained to me carefully how I should take the pills, how the Chagas came and what we should take care of. (…) Now I am healthy. Now I am calm.” (Severina, family 1)*.

*“My sister was already doing the treatment. (…) She was giving more importance to Chagas disease. (…) Almost together, we did the treatment. She supported me more than anyone did. She has finished the treatment. The doctor told me, ‘Bring your daughters, we’ll do the tests.’ So, I brought them.” (Marta, family 3) “Yes, my husband is also doing the treatment. He is doing it in Spain. In Barcelona.” (Marta’s sister; family 3)*

**Fig 4:**
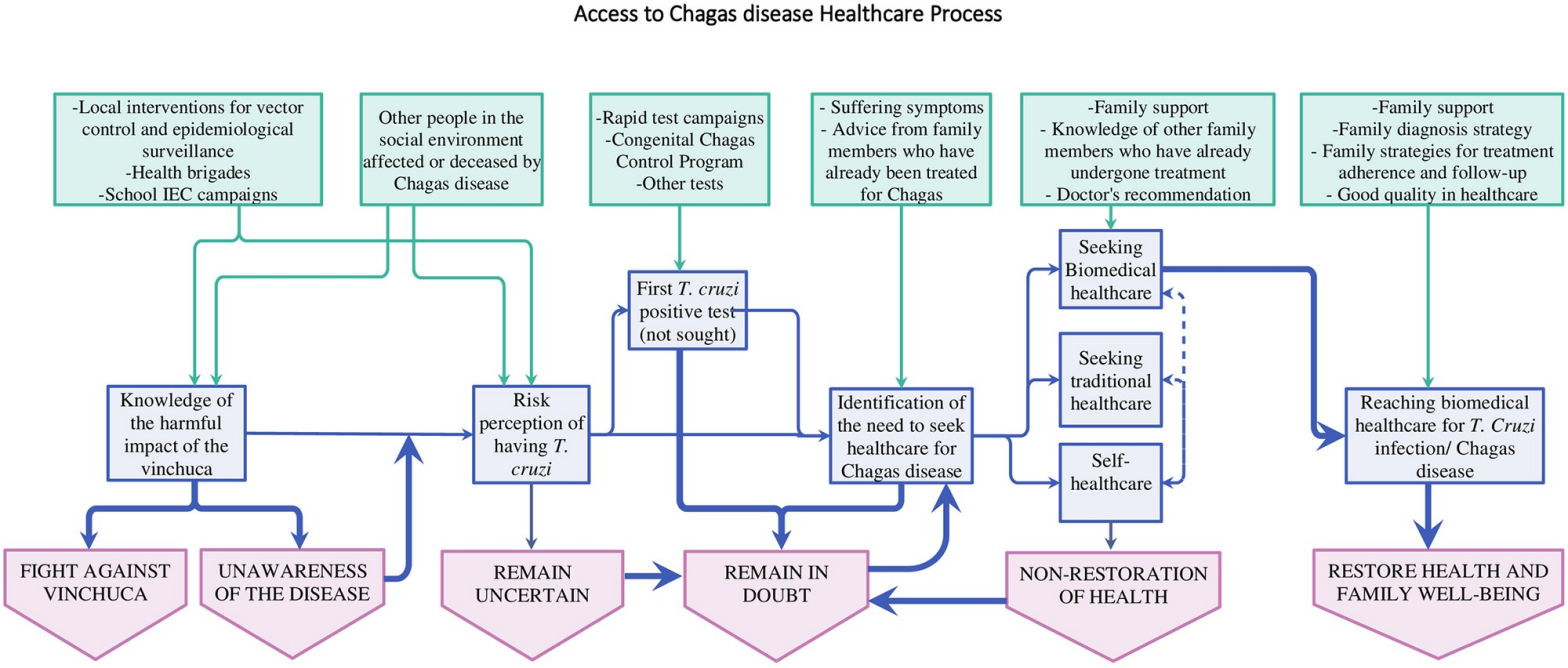
Decision-making in the access to health care for Chagas disease. In green, the most important facilitators that influence the entire process of decision-making in seeking and reaching health care for Chagas disease. In blue and purple, the steps of the common route in the decision-making.

By observing the timelines for each biographical history, the process to access to biomedical health care for Chagas disease noticeable attains a very long-term goal and proceeds through several steps involving unawareness, uncertainty, and doubts influenced by the lack of accessibility to health care along their lifetime. However, stories also reveal that other people decided to remain in these positions due to fears, myths, lack of awareness, or inability to access the biomedical health care system. See the whole process synthesizes in Fig 4.

## Discussion

Research subjects recalled, assessed, and communicated the complexities of Chagas disease from a systematic structural understanding of the context in which they live and from their personal and familiar experiences. They described environmental and social characteristics of the context that entails exposure to the vector and diverse social inequalities that highlight the marginalization and socioeconomic vulnerabilities that research subjects have suffered. Similar results were found by Forsyth C. in Santa Cruz, Bolivia, where Chagas disease is perceived as undoubtedly related to marginalization and poverty in the countryside [56]. These meanings are also identified by the scientific and biomedical community as risk factors for exposure to the vector and consequently for having *T. cruzi* infection. Communication and clinical practices are often reduced to these factors and Chagas disease is stereotyped as a poverty-related disease. This causes stigmatization and discrimination, which limits the detection of the disease in all socioeconomic classes [59].

Social representations influence people’s attitudes and actions in their daily lives, constituting a common reality in the ways of facing (or not) Chagas disease. However, these cognitive elements have a dynamic development as interpreted by individuals [73]. Families in the study have lived uninformed about Chagas disease for years, and the silent nature of the infection left it unnoticed. Lack of information and misinformation about the antiparasitic treatment are linked to a prevalent fatality view, which is strongly established within all population groups residing in the Valle Alto, where historically low scientific evidence and deficient capability of the health system prevail. Fear, mistrust of the treatment, lack of confidence in the health system, and stigmatization justify passive positions among people who end up living with uncertainty and evading the problem. Sanmartino M. et al. explained the resignation as a response to the lack of assistance from the health system; within this context, a positive diagnosis would be a death sentence [11]. In addition, this study revealed the importance of avoiding the psychological effect of diagnosis on the progression of the disease. Forsyth C. found the same notion in Santa Cruz, Bolivia, where people prefer to maintain a state of emotional calm *(tranquilidad)* not to worsen symptoms [75]. In contrast, this study found very opposite perceptions within the same families, such as the denial of the existence of Chagas disease associated to disabled, sick, and/or elderly people. This is the first time that the authors have heard of skepticism about the existence of the disease with such clarity, and no reports of it have been found on the literature. On the other hand, associations with weakness and the elderly do coincide. For this same reason, a positive diagnosis of *T. cruzi*, accompanied by symptoms or not, also impacts on the social and work environments due to stigmatization and discrimination [11, 12, 76]. Nevertheless, there are also life stories that overcome all the obstacles and resistance to diagnosis and treatment thanks to family support and quality medical assistance. The way transnational families share information and support is also highlighted. A transnational approach to health care, such the Bolivian Chagas Platform, offers many opportunities to access health care *here and there* and to monitor the disease throughout the migration process [50].

Social groups generate specific knowledge and practices to prevent and face suffering. As documented in this study, they do so by using pluralistic health care systems. Selection within them is dynamic and non-exclusive, and depends on the type of illness, accessibility, and trust [67, 74]. Families take care of their members’ health and solve diseases. Self-care system through ethnomedicine is very common and was deeply described by several authors [77–79]. On other occasions, research subjects resorted to traditional or popular medicine and diagnosis of *witchcraft* (Brujería in Spanish, Laykasqa in Quechua). Experience of researchers in intercultural health in Bolivia and literature helped to understand better the bewitchment (Embrujo or Hechizo) according to the Aymara-Quechua conception [80–82]. The etiology is due to an action of a witchcraft done or ordered to be done by an enemy or envious person. The witchdoctor (brujo in Spanish, layqa in Quechua), is the person who cast an *embrujo* with magical forces. The syndromes, and conditions of the *embrujo* can be very diverse. In general, it brings bad luck to the affected person and his/her family. The person suffers from decay, social problems and isolation, and if the person is not timely treated it leads to death [80–82]. The subjects of the study reported some family cases with physical ailments produced by *embrujo*. Later, they confirmed that these manifestations corresponded to Chagas disease, a vector-borne disease. The diagnosis of *embrujo* is performed by a healer (curandero), a general term that refers to Yatiris, in Aymara, Jampiris in Quechua, or Kallawayas, itinerant healers-naturists from the province of Bautista Saavedra [80–82]. The *curanderos* typically represents the sage that mixture sorcerer, fortune teller and doctor (brujo, adivino and médico, in Spanish), and they use medicinal plants and the divination with coca leaves. The treatment tries to remove the supernatural force from the patient’s body with a magical preparation that annuls or reverses the effects of witchcraft and/or to return the evil to the person who provoked the *embrujo*. Likewise, the bewitchment can be prevented by not giving reason to enemies and not causing envy [80–82].

Dell’Arciprete A. et al. described a similar notion from the Pilaga community in Argentina, as an illness intentionally produced by another person or spirit [83]. In northern Bolivia, Bastien J.W. found that Chagas disease was described as *chuyma usu* (heart disease) and *empacho* (digestive problems due to enlarged bowels). Regarding popular beliefs, both mega-syndromes are caused by the breakdown of the balance in social or environmental relationships that underlie the conception of Andean health-disease [78]. Vandebroek I. et al. described the relationship with the *madre* (intestinal volvulus) and the heavy labor in agricultural fields in Capinota, Cochabamba [77]. One of the research subjects gave credit to the traditional therapists’ knowledge, as did Bastien J.W. in highlighting the value of Andean traditional healers from northern Bolivia in providing lessons on the environmental conditions that nurture the disease cycle [78]. Bolivia’s ethnocultural systems are very heterogeneous, converging, and intermingling. Intra-ethnic discrimination is driven by socioeconomic and cultural status differentiation. Research subjects from the three families expressed their indignation at the discriminatory treatment received by health personnel in hospitals. Dell’Arciprete A. et al. also described discriminatory attitudes toward the Pilaga and Wichi communities in Argentina [83].

The Unified Intercultural Community and Family Health System (SAFCI for its acronym in Spanish) is the intersectoral health policy with a community and intercultural family approach of the Plurinational Program of the Bolivian Government. The SAFCI is based on health promotion, disease prevention, and community participation. It recognizes traditional health and opens spaces in first-level facilities for traditional therapists. However, in the Valle Alto region, this does not appear to be very common and the families in the study revealed that they visit therapists outside biomedical fields. The intercultural approach implemented in Bolivia is criticized by several authors as insufficient and remarks the necessary conjunction of medical and traditional systems and their integration with inequalities and socioeconomic structural conditions for the establishment of truly respectful and symmetrical relationships [58, 67, 84]. The lack of decentralization and universalization of the public health system excludes impoverished populations that are far from urban centers and that did not request exclusive traditional care. On the other hand, the SAFCI’s familiar angle shows great potential. The SAFCI program participates in the Bolivian Chagas Platform program by bringing information to the communities. If the SAFCI personnel were trained, they would attend door-to-door Chagas disease follow-ups or pursue other participatory community actions [55]. Other family-based actions for the detection and health care for Chagas disease could be generated or included in vaccination or family planning programs [45] such as the second systematic diagnostic test for congenital Chagas that coincides with the vaccination scheme for under one-year-old babies (Congenital Chagas Program, 2004–2011) [48]. Research subjects exemplify in their accounts how everything related to Chagas occurs within families: from vector fight toward doubts, opposing positions, and family caregiving strategies. Thus, the comprehensive attention approach entails accompaniment and care for mental health, as well as follow-up and guidance to affected adult chronic patients in the adaptation to daily life conditions. Family alliances are of crucial importance to cope with Chagas disease and achieve access to health care so that well-being is restored. Thus, this study provides useful and truly relevant results for the consideration of families not only as recipients of programs but also as main drivers of change.

The inclusion of a gender perspective in scientific studies and health programs has been highlighted in recent years [85], especially in the Sustainable Development Goals defined by the United Nations, as a means of achieving gender equality and empowering all women and girls by combating discrimination, unjust social norms, sexual and reproductive issues, poverty, political participation, and disadvantages in access to education and health care [86]. Neglected tropical diseases disproportionately affect women and are a significant source of disability and stigma among them [87]; hence, gender roles may influence access to medical care for Chagas disease. This study shows that women are more motivated to participate and recognize the problem, thus highlighting the fundamental role of women in transmitting information and accompanying relatives to health care processes. However, strategies should include men’s involvement to share responsibilities and burdens and unravel unfair systemic gender roles. On the other hand, the difference in power between genders makes women subordinate to men’s opinion and authorization. This fact is relevant in the controversy about efficacy and tolerance of anti-parasitic treatment of Chagas disease. Therefore, as outlined in the PAHO and Doctors without borders’ guidelines, it is important to inform the patient and his/her family about the benefits and risks of treatment and to promote joint decision-making between the patient and the physician [16, 45], while encouraging family support.

The accessibility and quality of the public health system in Bolivia were questioned by research subjects, who demanded that it should be improved. In 2019, Law No. 1152 of the Single Health System (Sistema Unico de Salud—SUS) in Bolivia was approved and began to be implemented progressively [70]. The SUS promises that health care will be universal, free, and equitable and is based on effectiveness, comprehensiveness, interculturality, and intersectionality, among other principles. It is estimated that around 51% of the population will benefit from this service [88]. An appropriate regulation of the SUS law may be able to solve many of the current deficiencies found by this study. Somehow, decentralization and universalization of health care are necessary and must be accompanied by improvements in health administration, qualified health personnel, infrastructure, and resources, as well as by regulations of all forms of discrimination and violence in health-care facilities. In contrast, the care received at the Bolivian Chagas Platform was highly valued by families for being free of charge, administratively simple, with no queues, and of high quality. Pinazo et al., acknowledging the success of the Bolivian Chagas Platform and the scaling-up process, recommended the expansion of the Chagas Network of health facilities offering medical care for Chagas disease, more trained human resources, and closer monitoring of compliance with protocols [36, 50]. Thus, the *old paradigm* still so ingrained in the social and biomedical spheres could be deconstructed. Moreover, this study reveals a remarkable lack of information about Chagas disease beyond vector transmission and established myths in all sectors of society in the Valle Alto region. Educational actions need to demystify and destigmatize, as well as raise awareness, while providing access to comprehensive health care for all ages. Other authors have emphasized the impact of participatory educational perspectives that allow communities to analyze, decide, and lead prevention and health promotion actions [55, 89, 90]. Pardo et al. demonstrated the effectiveness and easily implemented viability of a Peer Education Program conducted in the Bolivian Chagas Platform in Punata [55].

On the other hand, although it is established by law, Chagas disease is not prioritized enough in hyper-endemic regions to ensure that the entire population has access to information and health care, to promote early detection and timely treatment at all ages, as well as social protection. Besides, testimonies show that actions for vertical control and campaigns for the detection of *T. cruzi* in pediatric ages should be reinforced. Bolivia, with the support of Belgian cooperation, has made this protocol mandatory after a pilot program (2004–2010) that demonstrated the effectiveness of early detection and treatment of newborns infected with the *T. cruzi* parasite [48]. However, according to observations in the Bolivian Chagas Platform, once external support for the Congenital Chagas Program ended (2011), the procedure began to operate irregularly in many of the public health services due to high staff turnover and the absence of supervision or support. Further research is needed to identify failures in the national control of the Congenital Chagas Program in Bolivia by interviewing women and the health personnel responsible for implementing its protocols.

Since municipal authorities are responsible for planning and prioritizing the financing of health interventions in first- and second-level facilities, it is advisable to intensify the coordination and monitoring of the processes together with SEDES, the ChNP and the Ministry of Health, as well as with other sectors, to comprehensively address Chagas disease [36, 50]. A project developed by the Global Chagas Coalition, ISGlobal, and the Illinois Institute of Technology has implemented an innovative tool (Xtrategy) in municipalities of the Bolivian Chaco region that strengthens dialogue and intersectional planning capacity to assess the impact and complexity of strategies and adequately select the interventions to be included in the municipal Annual Operating Plan [91].

The complexity of Chagas disease exceeds the biomedical component and must be addressed with an interdisciplinary approach with participation of all the stakeholders involved. Over the last decade, thanks to the common and collaborative effort of different stakeholders, including patient associations, academia, research community, and others, progress has been made in raising awareness of Chagas disease at the international level [33]. In addition, the International Federation of Associations of People Affected by Chagas Disease (FINDECHAGAS) has significantly increased the visibility of the disease and has recently succeeded in having the WHO designate April 14 as the International Chagas Day [92]. This study, immersed in a Science Shop process, claims to emphasize the social responsibility of science to generate evidence useful to solve the most important problems within communities. Furthermore, this work was demanded and nourished by the participation of ASCUCHAC, an organization with expertise in educating and raising awareness about Chagas disease and defending people’s rights.

### Limitations and biases

Qualitative research assumes a relative closeness of interpretations to a given phenomenon. The present study sheds light on people’s lives expressed by themselves. Nevertheless, the subjects’ narration of the social living conditions in the Valle Alto and the actions of the health system to address Chagas disease was completed with the literature to search for inconsistencies, contradictions, and exceptions.

This study might contain selection biases because there is a lack of variability in the characteristics of the families involved in terms of socioeconomic conditions and place of residence. The study went deeper into the life stories of families that reside in a hyperendemic context close to the nerve center of the Valle Alto. Although the research indirectly compiled the living reality of remote locations in the region, a more heterogeneous selection of families would have enabled the direct collection of data on inequalities between both contexts. There were limitations in locating families willing to engage as well as in reaching individuals within the selected families. Talking about such a stigmatized and socially silenced disease in the Valle Alto is really difficult. For people who have suffered the impact of the disease on their families, it means reliving painful moments. Others avoid or deny the existence of the disease. Thus, the study reached people who recovered their health and dared to tell their Chagas disease story.

The approach of the study did not contemplate collecting specific information on cases with chronic symptomatic infection that suffer cardiological and/or digestive disorders. Therefore, among the study subjects selected through hospital-based sampling and their relatives by the snowball technique, no person with this characteristic was found. Therefore, the results of this study did not reveal how people and their families are affected by living with advanced stages of the disease nor the specific access barriers to specialized care. The researchers encourage further research to shed light on the subject.

Since selection was made from the Bolivian Chagas Platform, biases may also arise due to the researchers’ relationships with the health system. Nevertheless, researchers gathered perceptions and experiences prior to contact with the Bolivian Chagas Platform, reviewing the entire history of their lives and triangulating data from different periods of time. Furthermore, memory biases may also be present. In any case, the process of triangulation among researchers mitigates observation and interpretation biases.

The study followed the Consolidated Criteria for Reporting on Qualitative Research (COREQ) [93]. The COREQ tool is a self-reflection checklist to perform a complete and transparent description of the study procedures and improve the rigor and credibility of the interviews and discussion groups under the qualitative methodology [93]. Thirty out of the thirty-two COREQ criteria were met. COREQ point 23, which indicates providing transcripts to the interviewees, was not applicable because of some of the interviewees’ low reading literacy. Nevertheless, phone calls were made to them to complete information or to resolve some of the researchers’ questions. COREQ point 28, which refers to interviewees providing feedback on the findings, was not applied, but conclusions and materials were discussed with the civil society group from which this bottom-up emerged. Further explanation of the analysis procedure is provided in S6 File.

### Highlights

1. Structural social determinants shed light on health vulnerability and inequality. Findings can guide the whole range of political sectors and levels to coordinate and mitigate them. The development of intersectoral actions requires the commitment of municipal authorities and the generation of deliberative spaces together with civil society to collectively design the best solutions to address Chagas disease.
2. There is a heterogeneity in the social representations of Chagas disease and the positions adopted to cope with it. As a special finding of this research, very opposing perceptions were described within the same families, such as the denial of the existence of Chagas disease associated to disabled, sick, and/or elderly people. Nevertheless, the association of Chagas disease with a fatal, incurable, disabling, and stigmatizing disease is widespread in all sectors of society in the hyperendemic region of Valle Alto. These notions have been built up over the years since the discovery of the disease due to global neglect, the delay in scientific research to obtain better diagnostic and therapeutic responses, the inability of the National Health System to provide health care, and the perpetuation of the old biomedical paradigm of Chagas disease.
3. The search for medical attention generally arises from the appearance of symptoms and the suspicion of having Chagas disease. Often a positive diagnosis generates huge mental health and social consequences. A comprehensive approach and early and timely management of Chagas disease are important to prevent the illness and reduce all physical, psychological, social, and occupational impacts.
4. Plural health-care systems allow the promotion of new health programs following the population’s own patterns, as a starting point for bringing the biomedical sphere closer to social reality without imposing scientific reasoning. However, health systems must be complementary to each other and, therefore, decentralization and universalization of the biomedical system is necessary as a first step to solve health inequalities.
5. Stigmatization leads to the communication about the disease only within families, leaving it socially invisible. A family approach and the opening of spaces that allow their commitment will promote circuits of existing information and support within families and relatives. In transnational spaces, families who migrated or with members who migrated also generate changes that go beyond borders.

## Conclusion

This study presents a Science Shop process to deliver scientific results based on the needs expressed by civil society groups and ASCUCHAC in the Valle Alto of Cochabamba, a hyperendemic area for Chagas disease. It focuses on life stories, bringing together families from Punata and Cliza that revealed social determining factors in the search and access to health care for Chagas disease. Effectively tackling Chagas disease requires intersectoral policies to confront also non-biomedical domains and to understand the intricacy of the problem with all its causes and implications. The participation of civil society and people who live the reality in policymaking is an ethical duty and ensures policy sustainability. Therefore, strategies need to target the challenges within the psychosocial field involving very opposing perceptions inside families, such as the denial of the existence of Chagas disease, and imperatively deal with structural inequalities. Likewise, changing the current social representations of Chagas disease requires a substantial improvement in accessibility to the public health system and comprehensive health care. Educational programs on Chagas disease must be accompanied by actions that guarantee access to health care for people of all ages, strengthening early detection in the acute phase and in the undetermined chronic phase as a strategy to provide timely antiparasitic treatment and prevent the illness and all its repercussions. Our findings are transferable to the entire Valle Alto region and other hyperendemic areas of Bolivia. The approach is replicable in other endemic and non-endemic locations, and it is highly recommended to understand the specific needs and particularities of the affected or potentially affected people and design context-specific strategies. Furthermore, the transdisciplinary approach allowed obtaining useful findings for ASCUCHAC, with translational and transformative potential to create deliberative environments in which families are not forced to keep their struggles and actions in secret and where they can openly talk about their fears and apprehensions to bring people living with *T. cruzi*/ Chagas disease out of oblivion.

## Supporting information

S1 File Transformative Science Shop process. (PDF)

S2 File In-depth interview guide (translated into English). (PDF)

S3 File Pre-categorization. (PDF)

S4 File Category tree. (PDF)

S5 File Informed consent (translated into English). (PDF)

S6 File Analysis procedure. (PDF)

## Abbreviations

ADRs: Adverse Drug Reactions
ASCUCHAC: Association of Corazones Unidos por el Chagas de Cochabamba
CEADES: Foundation for Science and Applied Studies for Health Development
ChNP: Bolivian Chagas National Program
InSPIRES: Ingenious Science Shops to promote Participatory Innovation, Research and Equity in Science
ISGlobal: Barcelona Institute for Global Health
NGOs: Non-Governmental Organizations
PAHO: Pan American Health Organization
SAFCI: Unified Intercultural Community and Family Health System
SEDES: Departmental Health Service
SUS: Single Health System (Sistema Único de Salud)
*T. cruzi*: *Trypanosoma cruzi*
WHO: World Health Organization
