# Supplementary material for: Social determinants in the access to health care for Chagas disease: A qualitative research on family life in the “Valle Alto” of Cochabamba, Bolivia
Source: PLoS One. 2021 Aug 12;16(8):e0255226. doi: 10.1371/journal.pone.0255226 (PMC8360591; doi:10.1371/journal.pone.0255226)
Supplement: S1 File — (PDF) [file pone.0255226.s001.pdf]

Living with Chagas: a qualitative study based on family stories in the Valle Alto of Cochabamba (Bolivia)

Science Shop Process

Science Shops are knowledge intermediary structures originally university-based to initiate research projects on topics defined jointly with civil society groups to generate transformative evidence on those needs or priorities. However, there are many different models of science shop and participatory research. The essential common characteristics are research generated from the bottom up and the exercise of scientific communication to strengthen the links between science and society.

The *Science Shop process* proposed by InSPIRES is a flexible framework that draws on the various models of science shop, Community-Based Participatory Research and the principles of Responsible Research Innovation, open science and impact assessment. This framework can be adapted to the context and needs of research topics according to their complexity (Urias et al. 2020).

The Science Shop process conducted in the present research work followed the below steps.

1. Collection of social demands or needs from a civil society group. See more about this phase in this short documentary video.
2. Reformulation of demands into research question.
3. Design and implementation of the research project
4. Returning the findings to the civil society involved
5. Disemmination of results and materials

Furthermore, over the course of the Science Shop process, it is implemented an assessment of short-term, mid-term and long-term impacts through the InSPIRES evaluation online-tool at the InSPIRES Open Platorm (Gresle et al. 2019).

"The tool delivers back a set of pieces of information through different visualizations which analyze each project's process in five dimensions, selected-constructed after a careful revision of public engagement and impact evaluation criteria proposed by different projects and researchers. The dimensions evaluated by this online instrument are: (i) Knowledge Democracy, (ii) Citizen-led Research, (iii) Participatory Dynamics, (iv) Integrity, and (v) Transformative Change. InSPIRES Open Platform (OP) becomes an open repository that allows comparison among participatory projects." (Gresle et al. 2019)

Gresle, Anne Sophie, Anna Cigarini, Leonardo de la Torre Avila, Irene Jimeno, Franco Bagnoli, Herman Dempere, Mireia Ribera, Eloi Puertas, Josep Perelló, and María Jesús Pinazo. 2019. "An Innovative Online Tool to Self-Evaluate and Compare Participatory Research Projects Labelled as Science Shops or Citizen Science." Pp. 59–72 in *Lecture Notes in Computer Science (including subseries Lecture Notes in Artificial Intelligence and Lecture Notes in Bioinformatics)*. Vol. 11938 LNCS. Springer.

Urias, Eduardo, Floor Vogels, Seda Yalcin, Rosina Malagrida, Norbert Steinhaus, and Marjolein Zweekhorst. 2020. "A Framework for Science Shop Processes: Results of a Modified Delphi Study." *Futures* 123:102613.

This research project received finance from: Ingenious Science shops to promote Participatory Innovation, Research and Equity in Science (InSPIRES) H2020-SwafS-2016-1 / Nº Propuesta: 741677

Dissemination of results

The findings of the study and the resulting materials will be communicated by ASCUCHAC to actors in different sectors of Punata and Cochabamba involved in the issue. The aim is to strengthen the horizontal dialogue in order to find jointly appropriate and viable proposals. In the same way, new questions and problems may arise which will give rise to a new transformative science shop process.

Collection of social demands or needs from a civil society group

A participatory workshop was held in Punata on October 2017 with a group of civil society and representatives of the ASCUCHAC Association (a total of 22 participants) to gather demands and needs regarding Chagas disease. Using participatory techniques and the development of a problem tree, emerging questions were collected and prioritized together. See here a short documentary video about this phase.

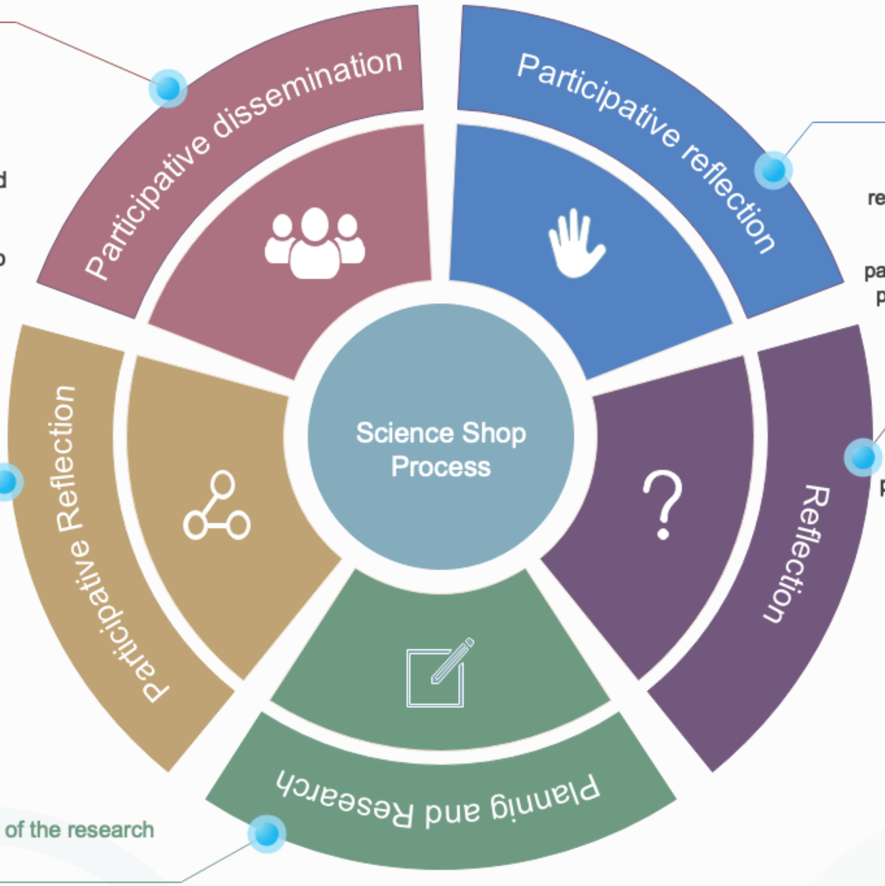

Returning of the findings to the civil society involved in the process

Findings were shared with ASCUCHAC and other community actors for discussion on the validity, usefulness and translatability of the results and materials. Likewise, a ficticious graphic story was offered to ASCUCHAC as a tool for communication, education and dialogue.

Reformulation in research questions

A scientific committee of the CEADES Foundation reformulated 57 social problems/demands collected in 84 research questions in October 2017. They were classified into 7 thematic categories. Then, another question is rescued from the list with the ASCUCHAC validation that raises the main research.

Design and Implementation of the research project

A joint research team was then established between the CEADES and ISGlobal institutions to design and implement the study. The interdisciplinary study based on family life stories was developed between November 2018 and March 2020.

This research project received finance from: Ingenious Science shops to promote Participatory Innovation, Research and Equity in Science (InSPIRES) H2020-SwafS-2016-1 / Nº Propuesta: 741677
