# Supplementary material for: Social determinants in the access to health care for Chagas disease: A qualitative research on family life in the “Valle Alto” of Cochabamba, Bolivia
Source: PLoS One. 2021 Aug 12;16(8):e0255226. doi: 10.1371/journal.pone.0255226 (PMC8360591; doi:10.1371/journal.pone.0255226)
Supplement: S2 File — (PDF) [file pone.0255226.s002.pdf]

Living with Chagas disease: a qualitative study based on family histories in the Valle Alto of Cochabamba (Bolivia)

Semi-structured interview guide

30-10-2018, translated version.

The objective is to describe the life history, chronologically, articulated around the subject of Chagas disease. These questions serve as a guide to the essential elements that underpin the realization of the entire process of health, disease and care of Chagas disease throughout the life of the study subject. The questions are formulated as triggers to open up topics of conversation and for the interviewee to develop his or her own experience with these topics. This script allows to detail step by step how decisions have been made regarding the different ways of dealing with Chagas disease and seeking medical care. This tool allows the interviewee to tell his/her story and prioritize the most important events and elements of his/her personal and family life. Thus, in the course of the interview, the questions can be reformulated and the sequence can be modified. The questions marked with (*) have been incorporated throughout the data collection process of the study.

**1. Presentation and collection of general characteristics**
-Age, Sex, Level of schooling*, Occupation/s, Language of preference*, Provision of health insurance, Current place of residence (locate the neighborhood), Description of the family members under the same roof. Description of the composition of the nuclear family, Description of their role in the family
**2. Recalling the childhood stage**
-Could you tell us about the place(s) where you were born and grew up?<br>- What was the house(s) where you were born like? With which family members did you share a home?<br>- What activities and occupations did your parents do, and what family activities did you participate in?*<br>- What did you know about the *vinchuca* at that time? Did you ever see it? Did other members of the family also detect *vinchuca* in the house or in the surroundings of the house?<br>- How would the family react when they saw the *vinchuca* and what they did subsequently?*<br>- Did you know anything about Chagas disease at that time?*
**3. Reviewing the adult stage.**
- Have you lived in other homes, in other places, or in other countries? How were those experiences and ways of living?<br>- Have you lived with *vinchuca* in your adult stage? Have you seen *vinchuca* in other places? Until what time have you seen *vinchuca*?<br>- When did you first hear about Chagas disease?<br>- How has Chagas disease appeared or interfered in your life? Do you know anyone who has Chagas disease?<br>- Have you ever thought that you might have Chagas disease? How appeared that thought and what did you do afterwards?*<br>-Have you ever received information about Chagas disease or have you been offered or suggested to do the Chagas test?*<br>- Have you ever thought about taking a Chagas test? When and why did you think about it? How did you feel about your health? What did you do next?
IF it is a MOTHER

This research project received finance from: Ingenious Science shops to promote Participatory Innovation, Research and Equity in Science (InSPIRES) H2020-SwafS-2016-1 / Nº Propuesta: 741677

- Were you tested for Chagas disease during your pregnancy(s) in the routine medical tests? How did you receive the diagnosis and what did you think and feel about it?
- (If positive on screening): Did doctors tested and followed up on the baby(s)?

4. Dealing with Chagas disease

IF DID the TEST

- Where did you take the test and how did you find the place to take the test?
- Do you know the result of the test? What were you told about it and what information were you given about Chagas?
- What did you think and how did you feel when you received the result? What was your first reaction after receiving the result of the diagnostic test and what decision did you make about it?
- Have you talked about your diagnosis with anyone in your environment?

IF TEST POSITIVE

- What decisions did you make to care your health and Chagas condition?
- Does having Chagas affect your daily life? In what way? Does it affect your family?

IF TEST POSITIVE and DID TREATMENT or FOLLOW-UP

- What made you decide to do the treatment or seek specific medical care for Chagas?
- How was the whole treatment experience like?

IF TEST POSITIVE and DID NOT TREATMENT or FOLLOW-UP

- How have you lived during this time knowing your diagnosis?*
- Were you informed about the options for treating Chagas disease and where to go? *
- Do you find it difficult to seek or reach medical care for Chagas disease?
- Is there anything that concerns you that is holding back you for treatment?

DID NOT the TEST.

- (If he/she doesn't see the need for the test). In what situation might you have thought you might have Chagas? In what situation would you think about taking the test? *
- (If he/she have decided to delay the diagnosis): What situation(s) have led you to delay seeking a diagnosis for Chagas disease?
- If those barriers described before did not exist, when and how would you seek testing? *
- If you had taken a test and it had been positive, what would you have thought about it and how would you have reacted?*

5. In the present

- How do you feel your health is at present?
- How do you imagine your future with Chagas / or the other people with Chagas? Can you/ they have a normal life?

This research project received finance from: Ingenious Science shops to promote Participatory Innovation, Research and Equity in Science (InSPIRES) H2020-SwafS-2016-1 / Nº Propuesta: 741677

Vivir con Chagas: un estudio cualitativo basado en historias familiares en el Valle Alto de Cochabamba (Bolivia)

Guión de entrevista semi-estructurada

Versión 30-10-2018

El objetivo es describir la historia de vida, de forma cronológica, articulada en torno al tema de Chagas. Estas preguntas sirven como guía de los elementos esenciales que sustentan la realización de todo el proceso de salud, enfermedad y atención de la enfermedad de Chagas a lo largo de la vida del sujeto de estudio. Las preguntas se formulan como disparadores para abrir temas de conversación y para que el entrevistado desarrolle su propia experiencia al respect. Este guión permite detallar paso a paso cómo se han tomado las decisiones en relación a las diferentes formas de enfrentar la enfermedad de Chagas y buscar atención médica. Esta herramienta sirve para que el/la entrevistado/a narre su historia y priorice los acontecimientos y elementos más importantes de su vida personal y familiar. Así, en el transcurso de la entrevista, las preguntas pueden reformularse y puede modificarse la secuencia. Las preguntas destacadas con (*) se han ido incorporando a lo largo del proceso de recogida de datos del estudio.

**1. Presentación y recopilación de las características generales**
- Edad, Sexo, Nivel de escolaridad*, Ocupación/es, Idioma de preferencia*, Provisión de seguro médico, Lugar de residencia actual (ubicar el barrio), Descripción de los miembros de la familia bajo el mismo techo. Descripción de la composición del núcleo familiar, Descripción de su papel en la familia.
**2. Recordar la etapa de la infancia**
- ¿Podría hablarnos del/ de los lugar/lugares donde nació y creció?<br>- ¿Cómo era la(s) casa(s) donde nació? ¿Con qué miembros de la familia compartió el hogar?<br>- ¿Qué actividades y ocupaciones realizaban tus padres y en qué actividades familiares participabas tú?*<br>- ¿Qué sabías de la vinchuca en esa época? ¿La viste alguna vez? ¿Otros miembros de la familia también detectaron vinchuca en la casa o en los alrededores de la misma?<br>- ¿Cómo reaccionó la familia al ver la vinchuca y qué hizo posteriormente?*<br>- ¿Sabías algo sobre la enfermedad de Chagas en ese momento?*
**3. Revisión de la etapa adulta.**
- ¿Has vivido en otras casas, en otros lugares o en otros países? ¿Cómo fueron esas experiencias y formas de vivir?<br>- ¿Has convivido con la vinchuca en tu etapa adulta? ¿Has visto vinchuca en otros lugares? ¿Hasta qué momento has visto vinchuca?<br>- ¿Cuándo oíste hablar por primera vez de la enfermedad de Chagas?<br>- ¿Cómo ha aparecido o interferido la enfermedad de Chagas en tu vida? ¿Conoces a alguien que tenga la enfermedad de Chagas?<br>- ¿Has pensado alguna vez que podrías tener la enfermedad de Chagas? ¿Cómo apareció ese pensamiento y qué hiciste después?*<br>-¿Has recibido alguna vez información sobre la enfermedad de Chagas o te han ofrecido o sugerido hacer la prueba de Chagas?*<br>- ¿Has pensado alguna vez en hacerte la prueba de Chagas? ¿Cuándo y por qué lo pensaste? ¿Cómo te sentiste respecto a tu salud? ¿Qué hiciste después?
SI es una MADRE

This research project received finance from: Ingenious Science shops to promote Participatory Innovation, Research and Equity in Science (InSPIRES) H2020-SwafS-2016-1 / Nº Propuesta: 741677

- ¿Te hicieron la prueba de la enfermedad de Chagas durante tu(s) embarazo(s) en las pruebas médicas rutinarias? ¿Cómo recibió el diagnóstico y qué pensó y sintió al respecto?<br>- (Si es positivo en la prueba de detección): ¿Los médicos le hicieron pruebas y seguimiento al bebé(s)?
**4. Abordaje de la enfermedad de Chagas**
SI HIZO la PRUEBA
- ¿Dónde hiciste la prueba y cómo encontraste el lugar para hacerla?<br>- ¿Sabes el resultado de la prueba? ¿Qué te dijeron al respecto y qué información te dieron sobre el Chagas?<br>- ¿Qué pensaste y cómo te sentiste cuando recibiste el resultado? ¿Cuál fue tu primera reacción tras recibir el resultado de la prueba diagnóstica y qué decisión tomaste al respecto?<br>- ¿Has hablado de tu diagnóstico con alguien de tu entorno?
SI LA PRUEBA DA POSITIVO
- ¿Qué decisiones tomaste para cuidar tu salud y la condición de Chagas?<br>- ¿Tener Chagas afecta a tu vida diaria? ¿En qué sentido? ¿Afecta a tu familia?
SI LA PRUEBA DA POSITIVO y SE HACE EL TRATAMIENTO o EL SEGUIMIENTO
- ¿Qué te hizo decidirte a hacer el tratamiento o buscar atención médica específica para el Chagas?<br>- ¿Cómo fue la experiencia del tratamiento?
SI LA PRUEBA DA POSITIVO y NO SE HACE TRATAMIENTO o SEGUIMIENTO
- ¿Cómo has vivido este tiempo sabiendo tu diagnóstico? *<br>- ¿Te informaron sobre las opciones de tratamiento de la enfermedad de Chagas y dónde acudir? *<br>- ¿Te resulta difícil buscar o conseguir atención médica para la enfermedad de Chagas?<br>- ¿Hay algo que te preocupe y que te frene para el tratamiento?
NO HIZO LA PRUEBA.
- (Si no ve la necesidad de la prueba). ¿En qué situación habrías pensado que podrías tener Chagas? ¿En qué situación pensarías en hacerte la prueba? *<br>- (Si ha decidido retrasar el diagnóstico): ¿Qué situación(es) le ha(n) llevado a retrasar la búsqueda del diagnóstico de la enfermedad de Chagas?<br>- Si no existieran las barreras antes descritas, ¿cuándo y cómo buscaría hacerse la prueba? *<br>- Si se hubiera hecho la prueba y ésta hubiera sido positiva, ¿qué habría pensado al respecto y cómo habría reaccionado?
**5. En el momento actual**
- ¿Cómo crees que está tu salud en la actualidad?<br>- ¿Cómo imaginas tu futuro con Chagas / o el de otras personas con Chagas? ¿Puedes tener tú / ellos una vida normal?

This research project received finance from: Ingenious Science shops to promote Participatory Innovation, Research and Equity in Science (InSPIRES) H2020-SwafS-2016-1 / Nº Propuesta: 741677
