# Supplementary material for: Social determinants in the access to health care for Chagas disease: A qualitative research on family life in the “Valle Alto” of Cochabamba, Bolivia
Source: PLoS One. 2021 Aug 12;16(8):e0255226. doi: 10.1371/journal.pone.0255226 (PMC8360591; doi:10.1371/journal.pone.0255226)
Supplement: S3 File — (PDF) [file pone.0255226.s003.pdf]

Living with Chagas: a qualitative study based on family stories in the Valle Alto of Cochabamba (Bolivia)

Pre-categorization. Forecast of categories that can be found in the In-Depth Interviews

	THEMES	CATEGORIES	DESCRIPTION OF CATEGORIES
**STRUCTURAL COMPONENT**	**Structural Social Conditions**<br>Description of the structural root causes linked to the common living conditions of the social context.	Sources of economic development	Possibilities of the community of which the person interviewed has been a part to create wealth in order to maintain economic and social prosperity or well-being.
		Social context	Geographical, environmental, social, political and cultural characteristics of a space or environment, as well as the macro-structural relations and dynamics.
		Ownership and use of territory	Access to and use of natural resources, collective areas, and collective daily practices that allow for the satisfaction of social needs and community coexistence.
		Ethno-cultural Identity	Cultural identity is the feeling of belonging or identification with a community with a set of values, traditions, symbols, beliefs and modes of behavior of an ethnic and cultural nature that function as a cohesive element in the group.
		Health Equity	Analytical category to assess equity in health as the capacity of a social system to guarantee health resources to each individual according to his or her needs and to allow each individual to contribute in accordance with their abilities.
	**Familiar Life Conditions**<br>Descriptions of the family life conditions that can intervene in the collective family experience with Chagas disease.	Family Composition	The composition refers to the number of members forming the family unit and the different types of family units.
		Family Structure	It refers to the position and roles of each of the family members and the processes of interrelationship between them.
		Family economy	Family income that allows the satisfaction of their material needs, attending to fundamental aspects of their development and well-being.
		Family migration history	This category describes the process of family migration (as a whole or a member of the family) including internal, rural-urban, and outbound migration processes. In addition, the motivations and the impact on the family of the displacements are analyzed.
		Housing	Description of the characteristics of the residence, the area of the peri-domicile and the household activities that are developed.
	**Individual Life Conditions**<br>Descriptions of their living conditions that can act as dynamic	Gender	"Gender refers to the roles, behaviors, activities, attributes and opportunities that any society considers appropriate for girls and boys, and women and men. Gender interacts with, but is different from, the binary categories of biological sex. Different roles and

This research project received finance from: Ingenious Science shops to promote Participatory Innovation,
Research and Equity in Science (InSPIRES) H2020-SwafS-2016-1 / Nº Propuesta: 741677

	pressures in living with Chagas disease throughout their lives		behaviors can generate gender inequalities with respect to both their health status and their access to health care." WHO
		Level of Schooling	Level of schooling in the formal education system in Bolivia. The basic education consists of three levels: Preschool, Primary, and Secondary. Higher education includes university education and professional and technical training.
		Occupation(s)	Jobs, work or activities, paid or unpaid, that he currently undertakes, as well as other occupations that he may have performed throughout their lives.
		Life priorities	Description of the person's day-to-day priorities in relation to their interests, concerns or needs
	**Personal history about Chagas** Descriptions in relation to their experience with the vector and/or with Chagas disease.	Living with *vinchuca*	Experience and personal experience in relation to observation, contact or coexistence with *vinchuca* throughout your life.
		Diagnosis of *T. cruzi* infection	Circumstances leading to the detection or diagnosis of Chagas disease and experience around the laboratory analysis.
		Living with *T. cruzi* infection	Living with the knowledge of having *T. cruzi* infection or Chagas disease.
		Living the illness	category that describes the experience of suffering from Chagas disease (illness) and the prognosis for the future.
**PSYCHO-SOCIAL COMPONENT**	**Knowledge and perceptions** Notions, experiences, perceptions, beliefs and symbolic elements of Chagas disease	Lack of knowledge	Complete or incomplete lack of knowledge about Chagas disease.
		Identity of Chagas	Main characteristics of Chagas disease that are learned and communicated in the social environment. First descriptions that are thought of when hearing about Chagas disease.
		Attributable causes	Description of the causes attributed to Chagas disease.
		Risk factors	Description of the factors that increase the likelihood of exposure to having or suffering from Chagas disease.
		Physical consequences	Description by the person interviewed of the alterations or deviations in physiological state (signs and symptoms) as a consequence of Chagas disease.
		Psycho-emotional consequences	Alterations in health and well-being compromising the psychological and emotional state that affects individuals and how they relate to their environment.
		Family consequences	The impact on the composition, structure and family relationship produced by Chagas disease when it affects a family member.
		Social consequences	Consequences that mark the social and labor relations of the people affected, as well as the social contribution to the community.
		Risk perception of having *T. cruzi* infection	The ability to perceive the possibility of having *T. cruzi* infection or Chagas disease based on experience and knowledge of the causes attributable to Chagas.
		Communication of Chagas	Description of communication processes as the conscious action of exchanging information between two or more people in order to transmit or receive information regarding Chagas.
	**Attitudes in the search for Chagas disease healthcare**	Passive attitudes	It describes attitudes that are characterized by being passive or rejecting the search for medical care and/or the prevention of Chagas disease and the personal reasons underlying them.

This research project received finance from: Ingenious Science shops to promote Participatory Innovation,
Research and Equity in Science (InSPIRES) H2020-SwafS-2016-1 / Nº Propuesta: 741677

	Description of the person's attitudes as the learned or constructed predisposition that includes the affirmation of convictions and feelings about it and contributes to determining a variety of behaviors in relation to Chagas disease healthcare.	Active attitudes	It describes the active attitudes towards seeking medical care and/or preventing Chagas disease and the personal reasons underlying them.
**HEALTH SYSTEM COMPONENT**	**Actions to Chagas disease prevention, control and healthcare** Descriptions of the actions or practices carried out by the person interviewed, their family and/or their environment to prevent, control or care for Chagas disease.	Fight against *vinchuca*	Description of practices for vector control and responses to the presence of the vector.
		Biomedical Health Care Search	Description of the actions related to the search for Chagas disease Healthcare
		Medical self-care	Description of the actions and practices related to self-care of illnesses and Chagas disease, including self-diagnosis, self-treatment and self-medication.
		Search for popular or traditional health care	Description of the actions and practices related to health care through the traditional or popular system attending to traditional therapists for the prevention and care of diseases.
	**Access to biomedical healthcare for Chagas disease** Description of the events that facilitate or hinder the reach and use of biomedical healthcare services for the comprehensive care of Chagas disease.	Facilitators	Description of the factors that facilitate access to Chagas disease healthcare, including the strategies of individuals to reach these services, as well as the interventions and actions offered by the health system.
		Obstacles	Description of the factors that hinders the access to Chagas disease healthcare.
		Adherence	Actions to complete the diagnostic process and continue treatment and monitoring of *T. cruzi* /Chagas disease infection
		Consequences of medical use	Description of the effects or impact on the individual and family following the use of biomedical services for Chagas disease healthcare.

This research project received finance from: Ingenious Science shops to promote Participatory Innovation,
Research and Equity in Science (InSPIRES) H2020-SwafS-2016-1 / Nº Propuesta: 741677
