# Supplementary material for: Social determinants in the access to health care for Chagas disease: A qualitative research on family life in the “Valle Alto” of Cochabamba, Bolivia
Source: PLoS One. 2021 Aug 12;16(8):e0255226. doi: 10.1371/journal.pone.0255226 (PMC8360591; doi:10.1371/journal.pone.0255226)
Supplement: S4 File — (PDF) [file pone.0255226.s004.pdf]

Living with Chagas: a qualitative study based on family stories in the Valle Alto of Cochabamba (Bolivia)

Categorization tree

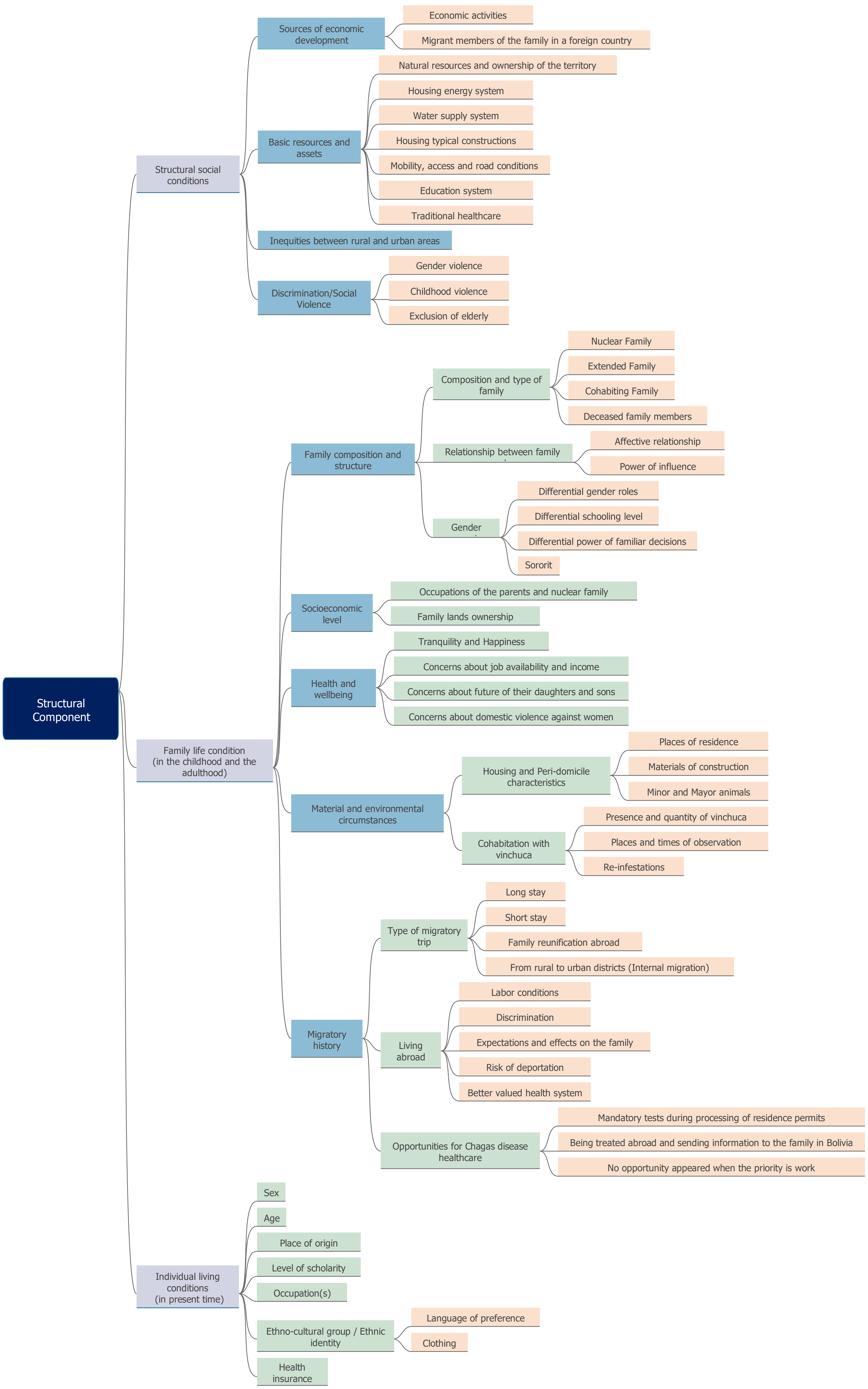

This research project received finance from: Ingenious Science shops to promote Participatory Innovation, Research and Equity in Science (InSPIRES) H2020-SwafS-2016-1 / Nº: 741677

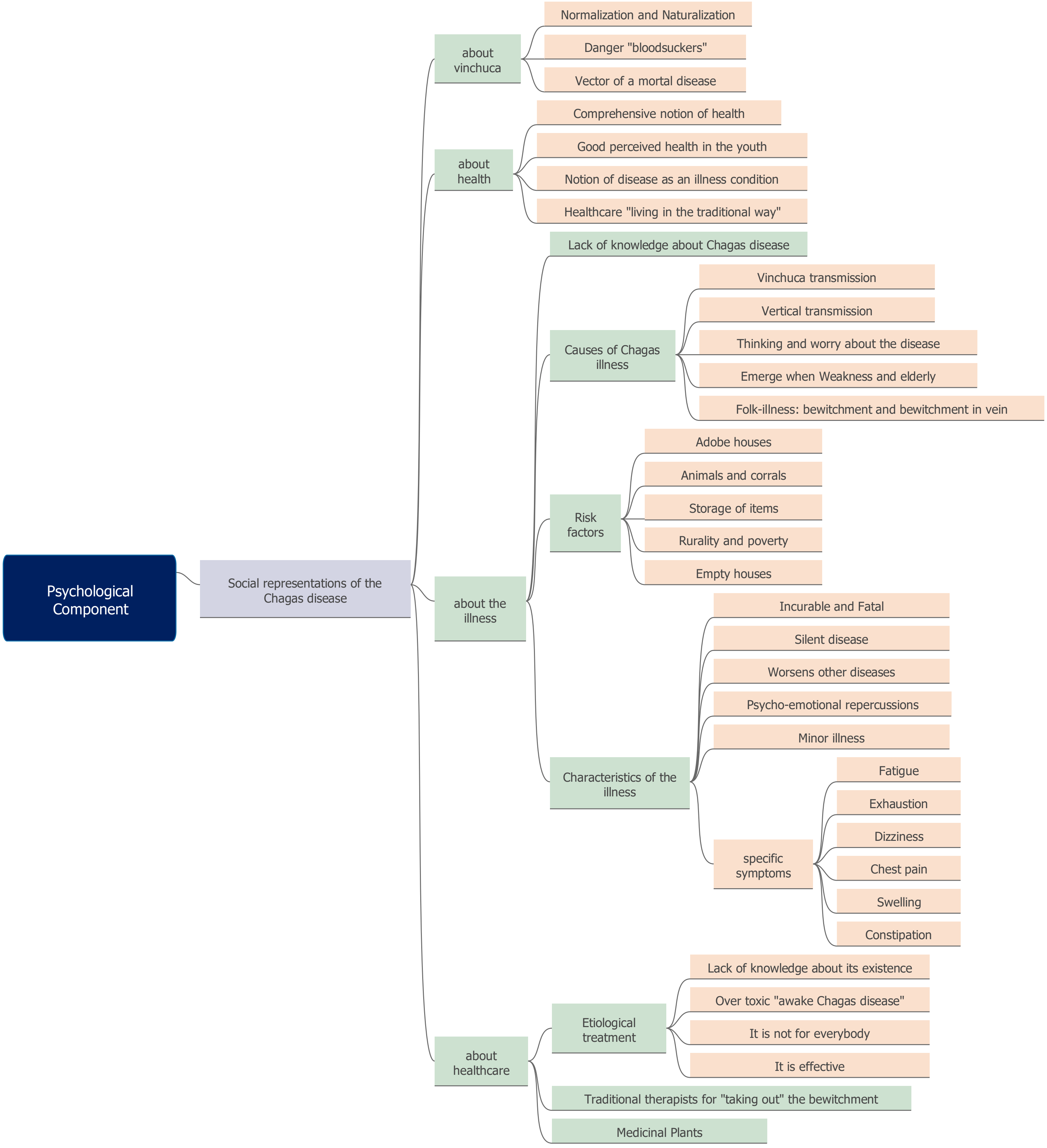

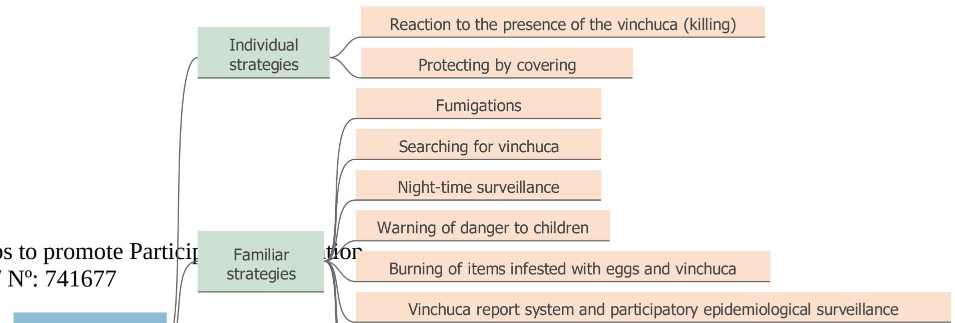

This research project received finance from: Ingenious Science shops to promote Particip[illegible]ion
Research and Equity in Science (InSPIRES) H2020-SwafS-2016-1 / Nº: 741677

This research project received finance from: Ingenious Science shops to promote Participatory Innovation, Research and Equity in Science (InSPIRES) H2020-SwafS-2016-1 / Nº: 741677

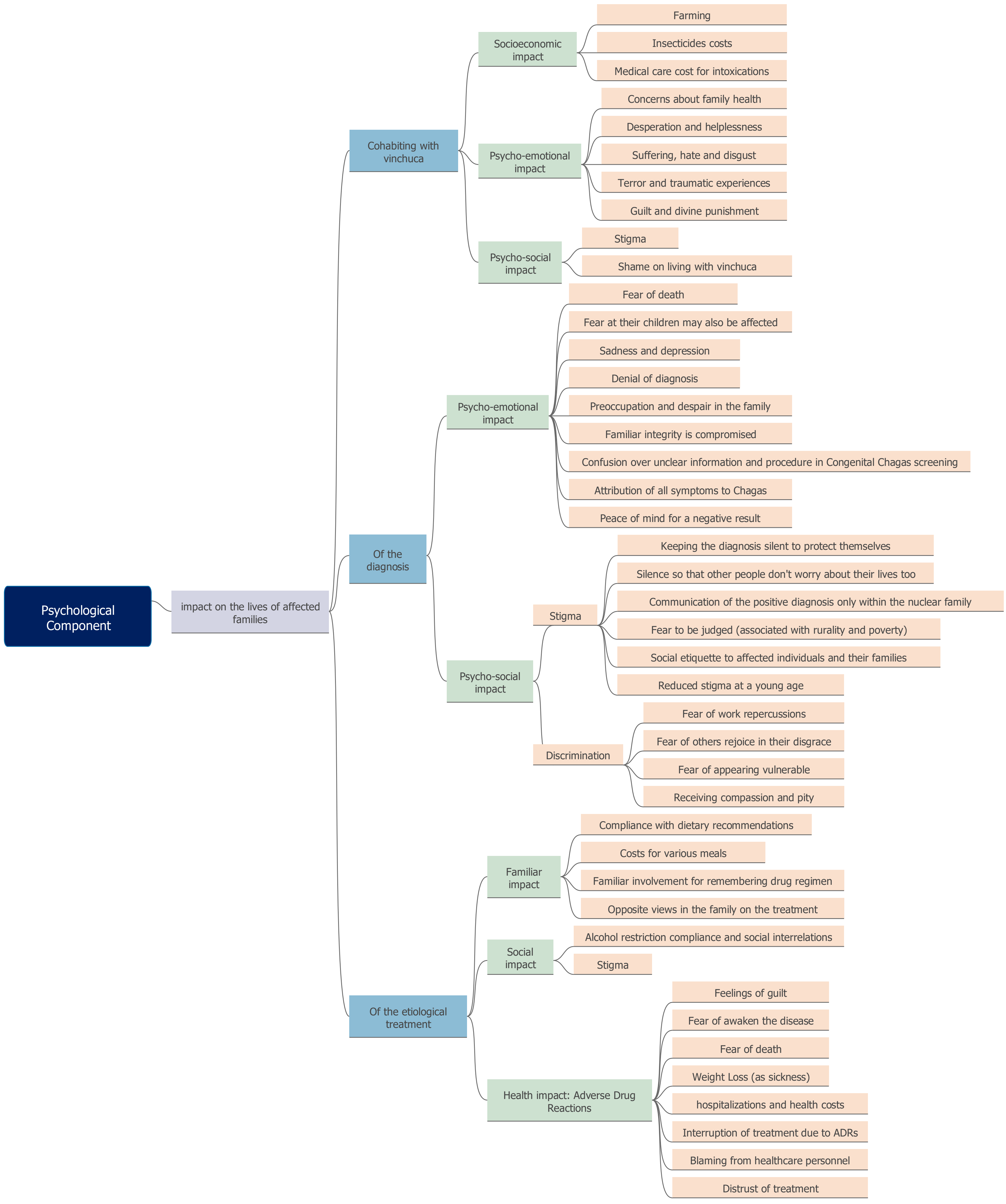

This research project received finance from: Ingenious Science shops to promote Participatory Innovation, Research and Equity in Science (InSPIRES) H2020-SwafS-2016-1 / Nº: 741677

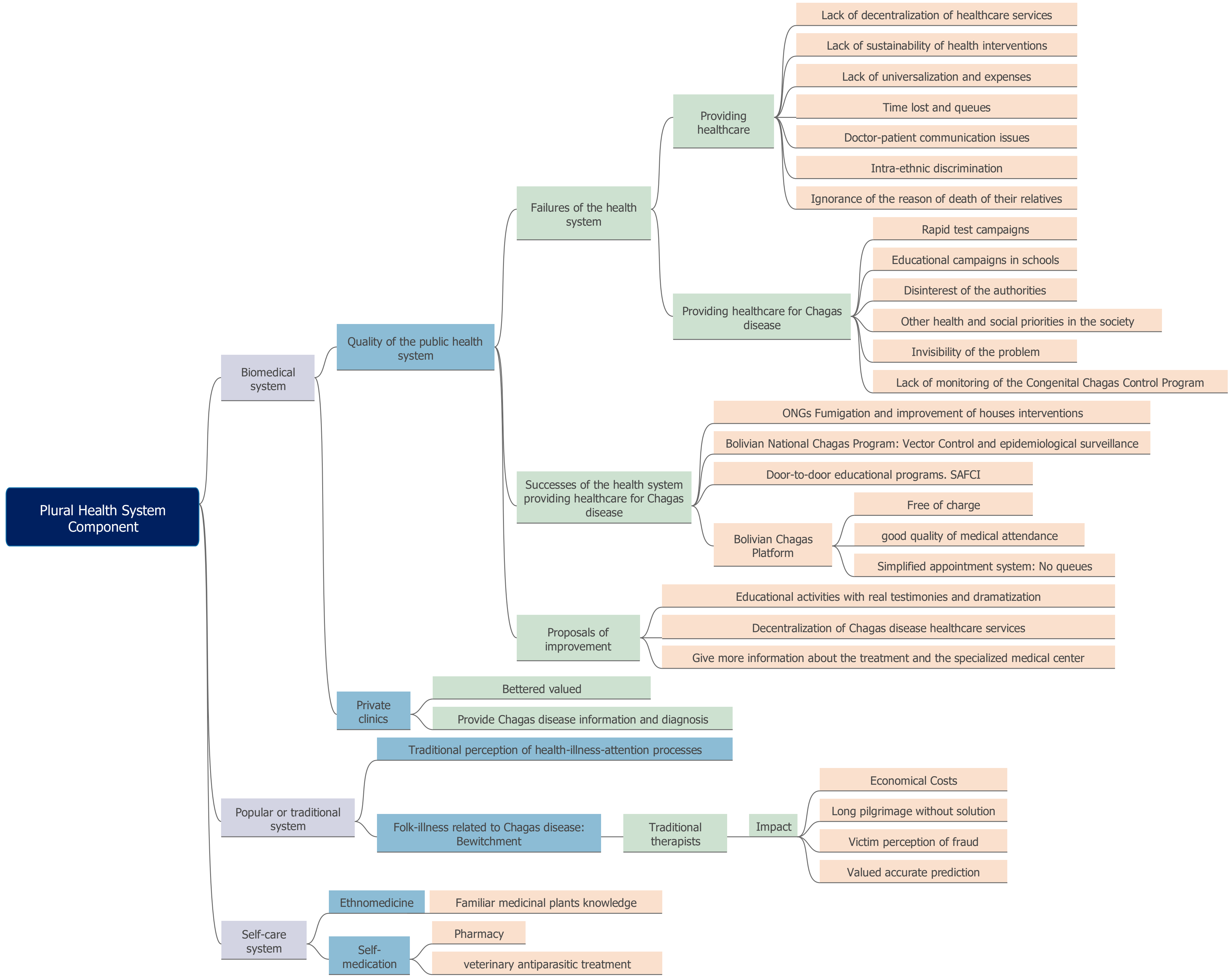

This research project received finance from: Ingenious Science shops to promote Participatory Innovation, Research and Equity in Science (InSPIRES) H2020-SwafS-2016-1 / Nº: 741677
