# Supplementary material for: Social determinants in the access to health care for Chagas disease: A qualitative research on family life in the “Valle Alto” of Cochabamba, Bolivia
Source: PLoS One. 2021 Aug 12;16(8):e0255226. doi: 10.1371/journal.pone.0255226 (PMC8360591; doi:10.1371/journal.pone.0255226)
Supplement: S5 File — (PDF) [file pone.0255226.s005.pdf]

Living with Chagas: a qualitative study based on family stories in the Valle Alto of Cochabamba (Bolivia)

Informed Consent Form

For research subjects

2018- version translated into English

Through this informed consent form we invite you to participate in research entitled: **Living with Chagas: A qualitative study based on family stories in the Valle Alto of Cochabamba**. This idea arose from questions formulated by the Community itself in a workshop held in coordination by the CEADES Foundation in October 2017, and is endorsed by the CEADES Ethics Committee.

The CEADES Foundation, together with ISGlobal from Barcelona Spain, has been working for several years to support the Bolivian Chagas Platform in Punata. Both institutions have seen the need for more research to better understand people's expectations and thus be able to improve healthcare for people affected by this disease, responding to the needs expressed by the community in Punata.

In this form we explain clearly what the research is about, what it is for, how and who will be executed and how subjects will involve.

What is the research about?

Several interviews will be carried out with patients of the Chagas Platform and their families to collect varied opinions about Chagas disease.

What is the purpose of this research?

Through this research we want to know: How do affected people and their families deal with Chagas disease in the Valle Alto of Cochabamba?

What decisions do they make between doing and not doing a Chagas analysis?

What actions do they take in the face of a positive result?

How does it impact on the family if one of the members has Chagas?

What factors prevent access to Chagas care centres?

Knowing all of this will allow us to see the person and understand better why people make certain decisions about Chagas. This will allow us to make recommendations to adapt health care to the needs of people with Chagas. In addition, this will help countries with a presence of Bolivian migrants to develop health strategies that include these aspects, hitherto unknown in the comprehensive care of Chagas.

How will their involvement be?

If you agree to participate in this study, you will be asked to tell your life story regarding Chagas disease and answer questions in several interviews lasting approximately one hour. What we discuss during these sessions will be recorded, so that the researcher can later transcribe the ideas you have expressed.

This research project received finance from: Ingenious Science shops to promote Participatory Innovation, Research and Equity in Science (InSPIRES) H2020-SwafS-2016-1 / Nº Propuesta: 741677

Voluntary participation and confidentiality

Participation in this study is voluntary, you will not receive any payment for participating. You may withdraw from the project at any time without harming your health care.
The information that is collected will be confidential and the data will be used in this study and similar studies. Your responses to the questionnaire and interviews will be coded using an identification number stored in the CEADES Foundation offices. Your name, address and personal data will not appear anywhere.

Signature of informed consent

I have read the information on this form or someone else has explained it to me in a language I understand well and all my doubts have been clarified.

I agree to voluntarily participate in this investigation in accordance with all the points that are written on this form.

I have also been told that I will have to answer questions in interviews, which will take approximately one hour.

Contact persons

If I have questions about my participation in this study, I can contact Dr. José Pedro Ribera Chávez at 4-4451676 of the CEADES Ethics Committee.

I understand that a copy of this consent form will be given to me, and that I can ask for information about the results of this study when it has concluded. For this, I may contact Dr. Daniel Lozano, at 4-4230009.

………………………………………………	………………………………..	………………..
Subjet Name	Signature (Fingerprint)	Date
………………………………………………	………………………………	………………….
Researcher Name	Signature	Date

In the case of illiteracy, the presence of an impartial witness is required throughout the informed consent process.

……………………………………………………	……………………………………	………………………
Witness Name	Signature	Date

This research project received finance from: Ingenious Science shops to promote Participatory Innovation, Research and Equity in Science (InSPIRES) H2020-SwafS-2016-1 / Nº Propuesta: 741677
