# Supplementary material for: Social determinants in the access to health care for Chagas disease: A qualitative research on family life in the “Valle Alto” of Cochabamba, Bolivia
Source: PLoS One. 2021 Aug 12;16(8):e0255226. doi: 10.1371/journal.pone.0255226 (PMC8360591; doi:10.1371/journal.pone.0255226)
Supplement: S6 File — (PDF) [file pone.0255226.s006.pdf]

Living with Chagas: a qualitative study based on family stories in the Valle Alto of Cochabamba (Bolivia)

Data Collection and Analysis Procedure

01

Planification and Pre-categorization

- Preliminary organization and planning among the interdisciplinary research team (four researchers from biomedical and social sciences) based on theory, research literature and empirical knowledge working with Chagas disease.
- Definition of an initial theoretical framework inspired by the Social Determinants of Health by the WHO and the Multidimensional framework for access to Chagas disease healthcare from Forsyth C. et al.
- Identification of initial themes according to the study objectives.
- Pre-categorization. Elaboration of an Initial Codebook for predicted categories.
- Elaboration of data collection tools: **In-Depth Interview Guide**
- The In-Depth Interview Guide was tested in a pilot interview to a patient of the Bolivian Chagas Platform in Cercado (Cochabamba), who agreed to collaborate voluntarily and consented orally.

02

Data collection

- Iterative telephone and personal contact with the collaborating families identified through facility-based sampling (Bolivian Chagas Platform in Punata) to arrange the interviews.
- Constant search for new collaborating individuals according to the selection criteria through snowball technique.
- Procedure for involvement in the study. Explanation of the **Informed Consent Statement Sheet.**
- Data collection through In-Depth Interviews led by a researcher and accompanied by two or three observers. Interviews were audio recorded with the consent of the interviewees.
- Three families and nine individuals were interviewed at their homes. Interviews lasted between 1h and 2h30.

03

Data transcription

- Transcription of the narratives, observation notes and non-verbal language.
- Translation narratives from Quechua into Spanish. (Meaningful expressions in the original language were preserved).
- Familiarization with data collected.

04

Coding and categorization

- Application of the initial codebook and inductive coding from the collected data. Manual and individual codification and categorization process by each researcher of the team following Qualitative content analysis including linguistics and interpretative analysis.
- Data triangulation with research literature and empirical data to find exceptions and contradictions.
- Triangulation among the interdisciplinary team of researchers to find coincidences and discrepancies, to redefine codes and categories names and to reduce observation and interpretation biases.
- Continuous collective reflection on the richness and thickness of data to achieve conceptual depth of the emerging categories and orientate the final number of individuals subjects of the study.

05

Structuration and theorization of results

- Comprehension and exposition of results to respond research questions by each researcher
- Triangulation among researchers
- Theorization of results building a **Resulting Theoretical Framework**

06

Interpretation

- Generation of conclusions and recommendations
- Presentation and discussion of the study findings with ASCUCHAC (Civil Society Organization) to reinforce the validity, transferability and usefulness of the findings.

This research project received finance from: Ingenious Science shops to promote Participatory Innovation, Research and Equity in Science (InSPIRES) H2020-SwafS-2016-1 / Nº Propuesta: 741677
